# Two-dimensional NIR-II AIE nanotheranostic probes with ultralarge Stokes shifts for surgical navigation and ablation of glioma

**DOI:** 10.1126/sciadv.aeb5389

**Published:** 2026-03-06

**Authors:** Yisheng Liu, Xiang Su, Yong Zhong, Ping Shangguan, Jun Zhu, Zhengqun Luo, Haigang Wu, Dong Wang, Ting Han, Jiefei Wang, Bingyang Shi, Ben Zhong Tang

**Affiliations:** ^1^ The Zhongzhou Laboratory for Integrative Biology, Henan International Joint Laboratory of Nanobiomedicine, School of Life Sciences, Henan University, Kaifeng, Henan 475004, China.; ^2^ School of Biomedical and Pharmaceutical Sciences, Guangdong University of Technology, Guangzhou, Guangdong 510006, China.; ^3^ Center for AIE Research, Guangdong Provincial Key Laboratory of New Energy Materials Service Safety, College of Materials Science and Engineering, Shenzhen University, Shenzhen, Guangdong 518060, China.; ^4^ Key Laboratory for Special Functional Materials of Ministry of Education, Henan University, Kaifeng 475004, China.; ^5^ School of Biomedical Engineering, University of Technology, Sydney, NSW 2007, Australia.; ^6^ Guangdong Basic Research Center of Excellence for Aggregate Science, School of Science and Engineering, The Chinese University of Hong Kong, Shenzhen, Guangdong 518172, China.

## Abstract

Near-infrared (NIR) fluorescence probes featuring ultralarge Stokes shifts and efficient aggregate-state luminescence are highly desirable for bioimaging yet remain scarce due to formidable synthetic challenges and intricate photophysical modulation. We report a facile one-step click reaction to synthesize a tetracyanoquinodimethane-derived NIR probe (TNQ2) that overcomes the long-standing nonfluorescence limitation while achieving an unprecedented 445-nanometer Stokes shift. TNQ2 self-assembles into the smallest-sized two-dimensional *J*-aggregates (sub-160 nanometers) with a red-shift absorption from 545 to 725 nanometers and aggregation-enhanced emission around 1000 nanometers. The radiative/nonradiative modulation balances the fluorescence/photothermal/photodynamic effect to support high-sensitivity NIR-II fluorescence imaging-guided precise glioma resection and postoperative phototherapy to substantially extend survival in orthotopic glioma models. Our molecule/nanoengineering strategy establishes a transformative paradigm for developing advanced NIR phototheranostics for brain diseases.

## Teaser

A 2D NIR-II AIE probe enables precise glioma surgery and postoperative phototherapy via an ultralarge Stokes shift.

## INTRODUCTION

Glioblastoma is a highly invasive central nervous system tumor with high mortality rate (*1*, *2*). Now, surgical resection is the primary clinical treatment (*3*) but heavily relies on precise tumor boundary identification by clinicians (*4*, *5*). The lack of highly sensitive, real-time intraoperative imaging technology often leads to incomplete glioma resection and high recurrence rates. Fluorescence imaging has emerged as a powerful nonradiative tool for tissue visualization (*6*–*8*), particularly near-infrared II (NIR-II) (1000~1700 nm) fluorophores (*9*–*11*), which stand out owing to their superior tissue penetration, suppressed autofluorescence, reduced phototoxicity, and enhanced sensitivity, making them ideal for precise surgical navigation and deep-tumor phototherapy (*12*–*15*). The construction of NIR-II fluorophores generally relies on introducing strong donor and acceptor units, extending π-conjugation, and strengthening structural rigidity to ensure NIR absorption and NIR-II fluorescence (*16*). Until now, various NIR-II fluorophores have been developed (*17*–*19*). However, most reported NIR-II probes suffer from small Stokes shifts (causing severe cross-talk between excitation and emission light), excessive π-π stacking (causing poor aggregate-state luminescence) (*20*), low signal-to-background ratios, and unsatisfactory therapeutic efficiency. In the past years, great efforts have been devoted to developing NIR-II emitters with large (>120 nm) or ultralarge Stokes shifts (>300 nm), but the existing examples still remain scarce (*21*, *22*). Moreover, the reported fluorophores often feature complex and bulky structures and require tedious, time-consuming, and costly multistep synthesis via transition metal-catalyzed stepwise coupling of multiple modules (donor, acceptor, π-bridge, etc.) (*23*–*25*). Therefore, developing simple NIR-II fluorophores with ultralarge tunable Stokes shifts, efficient aggregate-state fluorescence, high sensitivity, and strong phototherapeutic activity through facile and efficient synthetic strategies is highly desirable but remains an unmet challenge, especially for precise glioma theranostics.

In addition to molecular engineering strategies, the modulation of nanoassemblies through precise control of molecular arrangement (*26*), interlayer interactions, and morphology/dimensional confinement (*27*) effects serve as another powerful tactic to construct NIR-II materials with tunable photophysical properties. For instance, two-dimensional (2D) organic layered nanomaterials with tailorable planar geometries (*28*) provide an ideal photoconversion platform for biomedical applications, particularly in precise phototherapy of brain diseases characterized by complex central nervous system structures and pathology. Among them, 2D aggregation-induced emission (AIE) *J*-aggregates combined the advantages of *J*-aggregates and AIE materials and are promising candidates due to their large specific surface area, ordered molecular arrangement, flexible photoenergy modulation, and efficient aggregate-state luminescence (*29*, *30*). Compared to disordered aggregates or *H*-aggregates, *J*-aggregates with orderly slipped stacking (*31*, *32*) not only substantially enhance fluorescence intensity but also induce a bathochromic absorption shift (*33*–*35*). However, current fabrication strategies for 2D AIE *J*-aggregates, such as the adsorption of AIE molecules on 2D inorganic scaffold (e.g., black phosphorus) (*36*, *37*) or seeded growth-self-assembly (*38*), face critical limitations including complex exfoliation processes (*39*, *40*), low loading capacity, large size (1 to 100 μm), poor blood-brain barrier (BBB) penetration, and inefficient photoenergy utilization. These challenges highlight the urgent need for innovative molecular designs and nanoengineering approaches to develop 2D AIE nanosheets (NSs) with enhanced BBB permeability, large Stokes shifts, and balanced photoconversion efficiency for precise NIR-II surgical navigation and postoperative phototherapy of glioma.

**Fig. 1.**
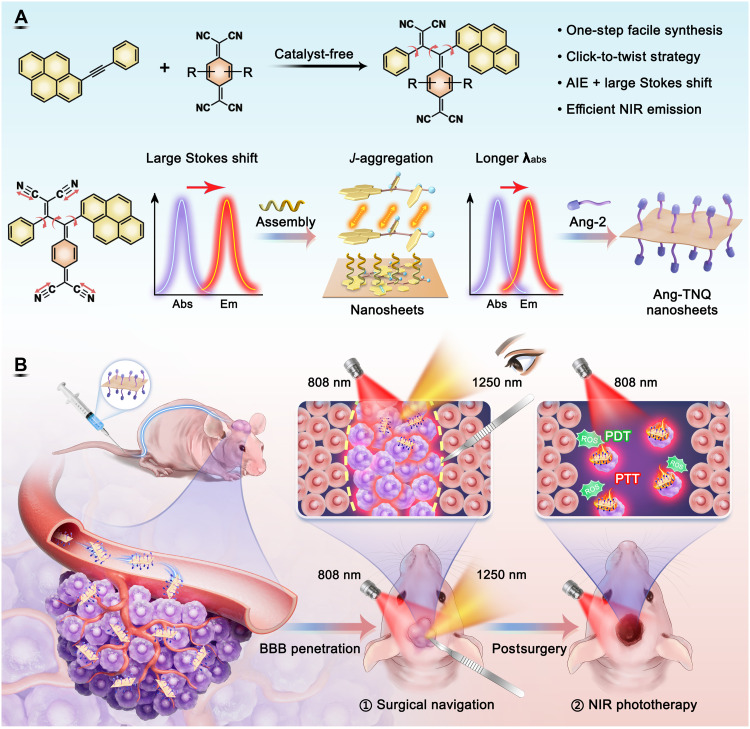
Scheme of 2D NIR-II AIE nanotheranostic probes with ultralarge Stokes shifts for surgical navigation and ablation of glioma. (**A**) Synthesis of TNQ molecules and their self-assembled NSs, accompanied by photophysical property changes. (**B**) Schematic illustration of the surgical navigation resection of orthotopic gliomas and postoperative ablation.

Herein, we developed an integrated cascade strategy combining click-to-twist structure modulation with controlled self-assembly to construct a 2D NIR-II AIE nanotheranostic system (Fig. 1A). Through catalyst-free [2 + 2] cycloaddition-retroelectrocyclization reactions between alkynes and electron-deficient 7,7,8,8-tetracyanoquinodimethane (TCNQ) or 2,3,5,6-tetrafluoro-TCNQ (F_4_-TCNQ), a click chemistry–based synthetic approach (*41*), we synthesized two donor-π-acceptor (D-π-A)–type AIE systems (TNQ1 and TNQ2) using pyrene-containing asymmetric internal alkynes, transforming planar reactants into highly twisted products. The resulting TNQ2 exhibited ultralarge visible-to-NIR-II Stokes shifts of up to 445 nm and exceptional antiquenching properties, overcoming the long-standing nonfluorescence limitation of TCNQ-based chromophores. The highly twisted TNQ2 molecules were further self-assembled into ultrathin 2D rectangular NSs (length/width: 160 nm by 110 nm) through controllable packing control, forming *J*-aggregates in the assemblies with a red-shifted absorption wavelength of 725 nm and enhanced fluorescence intensity to achieve better tissue penetration, photon absorption, and utilization. After Angiopep-2 (Ang-2) peptide modification, the Ang-TNQ2 NSs showed superior BBB penetration and glioma targeting capability, enabling precise NIR-II imaging-guided surgical navigation. The balanced fluorescence/heat/reactive oxygen species (ROS) generation under 808-nm irradiation effectively eliminated residual tumor cells through photodynamic/photothermal synergistic therapy (PDT/PTT; Fig. 1B). This work establishes the 2D NIR-II AIE system with an ultralarge visible-to-NIR-II Stokes shift, providing an advanced NIR platform for precise theranostics for brain diseases.

## RESULTS

### Synthesis and characterization of AIE luminogens

**Fig. 2.**
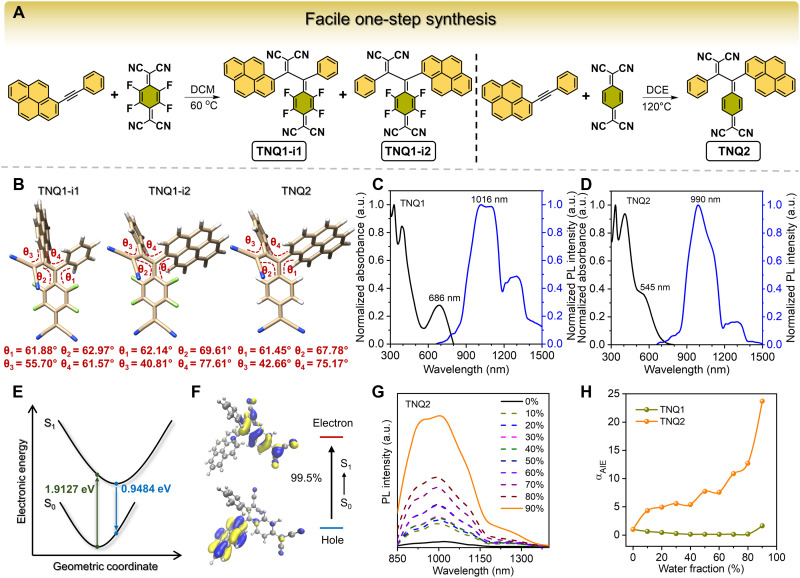
One-step synthetic routes and photophysical properties of TNQ-type luminogens. (**A**) Synthesis procedures of TNQ1 and TNQ2 via catalyst-free [2 + 2] cycloaddition-retroelectrocyclization reactions and their chemical structures. (**B**) Optimized S_0_ geometries (at the PBE1PBE/def2-tzvp level) and dihedral angles of TNQ1-i1, TNQ1-i2, and TNQ2. (**C** and **D**) Normalized absorption and PL spectra of TNQ1 and TNQ2, respectively. Excitation wavelength: 635 nm. a.u., arbitrary units. (**E**) Schematic illustration of the potential energy surface variation of TNQ2. (**F**) NTO plots corresponding to the S_0_→S_1_ electronic transition of TNQ2 calculated by TD-DFT (PBE1PBE/def2-tzvp). (**G**) PL spectra of TNQ2 in THF and THF/water mixtures with different water fractions. (**H**) Plots of the PL intensity enhancement (α_AIE_ = *I*/*I*_0_) of TNQ1 and TNQ2 versus the water fractions. Solution concentration: 100 μM.

The catalyst-free [2 + 2] cycloaddition-retroelectrocyclization reaction between alkynes and electron-deficient olefins has been reported to be a facile synthetic method toward chromophores with highly twisted skeleton, characteristic twisted intramolecular charge transfer (TICT), and long absorption wavelengths, but the products are generally nonfluorescent (*42*–*44*). To achieve NIR-emissive luminogens from such a facile click reaction tool, herein we designed and synthesized an asymmetric internal alkyne bearing a pyrene group and a phenyl substituent on the sides of carbon-carbon triple bond. The detailed synthetic route of the pyrene-containing alkyne was provided in fig. S1 according to a previous report (*45*). The introduction of the rigid, planar, and sterically hindered pyrene and the phenyl rotors around the highly twisted D-π-A backbone are expected to endow the products with enhanced fluorescence properties based on the restriction of intramolecular motion (RIM) mechanism and more tunable flexibility on the intermolecular interactions and self-assembly behaviors. On the basis of the abovementioned design principle, TNQ1 and TNQ2 were facilely synthesized through a one-step click reaction between the pyrene-bearing alkyne with F_4_-TCNQ and TCNQ, respectively, under heating conditions without the addition of any catalysts (Fig. 2A and fig. S2). Their structures were characterized by ^1^H nuclear magnetic resonance (NMR), ^13^C NMR, ^19^F NMR, and high-resolution mass spectrometry (HRMS). The results shown in figs. S3 to S7 revealed that TNQ1 existed as a mixture of two regioisomers, designated TNQ1-i1 and TNQ1-i2, which were very difficult to be separated, whereas TNQ2 was produced in a high regioselectivity. As expected, the optimized S_0_ geometries of TNQ1-i1, TNQ1-i2, and TNQ2 demonstrated highly twisted conformations with dihedral angles ranging from 40.81° to 77.61° (Fig. 2B).

Subsequently, the absorption and photoluminescence (PL) spectra of these TNQ compounds were investigated. As shown in Fig. 2 (C and D), the maximum absorption wavelength of TNQ1 and TNQ2 in dilute tetrahydrofuran (THF) solutions were 686 and 545 nm, respectively, with the corresponding molar coefficient (ɛ) value of 1.2 × 10^4^ and 1.1 × 10^4^ M^−1^ cm^−1^ (fig. S8). The normalized PL spectra of TNQ1 and TNQ2 revealed broad emission spectra covering the NIR-II window, with the maximum PL intensity peaked at 1016 and 990 nm, respectively. Noteworthily, TNQ2 showed an ultralarge Stokes shift of 445 nm, marking a substantial advancement over conventional organic dyes. To the best of our knowledge, such a large Stokes shift has not been documented in the literature. To elucidate the intrinsic mechanism of the large Stokes shifts, time-dependent density functional theory (TD-DFT) calculations were performed to analyze the molecular geometries, energy evolution between S_0_ and S_1_, and natural transition orbitals (NTOs) using the Gaussian 16 program package. Taking TNQ2 as an example, comparison of the optimized S_0_ and S_1_ geometries of TNQ2 suggested a distinct conformation change upon excitation (fig. S9 and tables S1 and S2). The dihedral angle between pyrene and the malononitrile-containing cyclohexadiene moiety in the S_0_ state of TNQ2 was measured to be ~61.45°, whereas in the S_1_ state, both groups adopted a nearly perpendicular arrangement with a dihedral angle of 83.10°. In addition, the dihedral angle between the phenyl plane and the malononitrile plane increased from 42.66° to 72.77° during the excitation transition. Further DFT calculations revealed that the simulated Stokes shift for TNQ2 is as high as 764 nm (fig. S10). As illustrated in Fig. 2E, the fluorescence emission energy of TNQ2 is 0.9484 eV, whereas the vertical excitation energy is 1.9127 eV, resulting in a large energy gap of 0.9643 eV. The NTO plots shown in Fig. 2F revealed the prominent charge separation between hole and electron, indicating that TNQ2 exhibited strong donor-acceptor charge transfer transition. Hence, the large Stokes shift may be rationalized by the pronounced difference in the equilibrium geometric structures between S_0_ and S_1_ states and the remarkable charge separation. Furthermore, the solvatochromic effect of TNQ2 was investigated (fig. S11), which showed that the Stokes shift of TNQ2 increased notably with solvent polarity. The Lippert-Mataga plot with a slope of 2.5 × 10^3^ indicated a substantial change in dipole moment between the S_0_ and S_1_, verifying that TNQ2 underwent efficient TICT upon photoexcitation and its emission mainly originated from a highly polarized and relaxed excited state. These results reflected that the large Stokes shift of TNQ2 may predominantly come from the reorganization or relaxation of the excited TICT state. Next, the PL behaviors of TNQ1 and TNQ2 were examined in THF/water mixtures to verify their AIE characteristics (Fig. 2G and fig. S12). As expected, these luminogens exhibited weak fluorescence as dispersed molecules in dilute THF solutions. As the water fraction gradually increased to 90%, the PL intensity of TNQ2 was remarkably enhanced by 23.7-fold, whereas the enhancement value for TNQ1 was only 1.6-fold (Fig. 2H). Given the larger Stokes shift, superior AIE properties, and well-defined structure, TNQ2 was selected for the subsequent investigation in morphology manipulation and biological applications.

### Manipulation in assembly morphology and photophysical properties

**Fig. 3.**
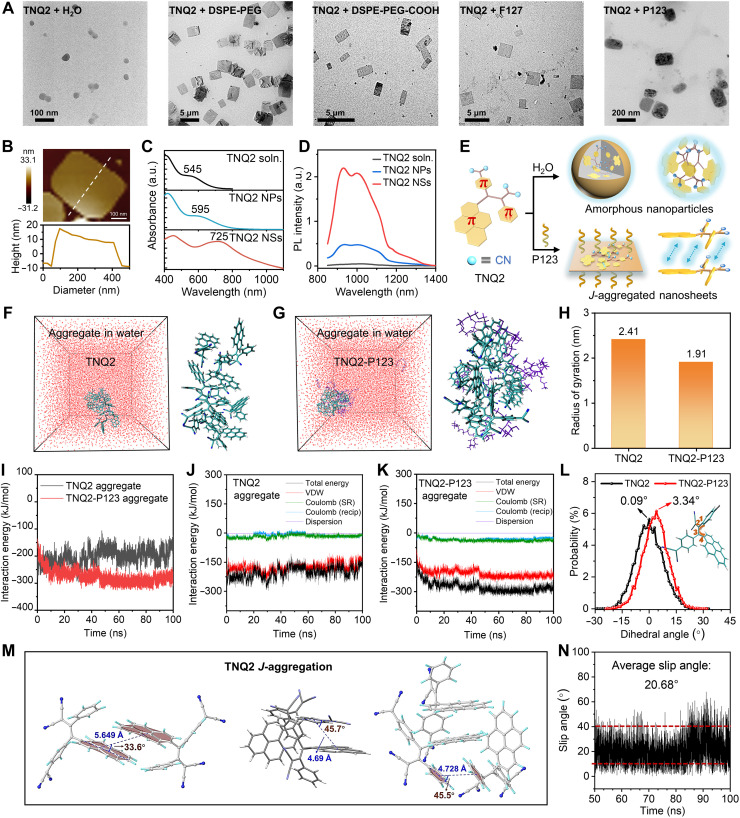
Morphological/photophysical adjustment and MD simulations of TNQ2 aggregates. (**A**) Transmission electron microscopy (TEM) images of TNQ2 aggregates prepared using different polymeric surfactants. (**B**) AFM image and the corresponding height profile of TNQ2 NSs prepared using P123. (**C** and **D**) Absorption and NIR emission spectra of TNQ2 monomer solution (soln.), TNQ2 NPs, and TNQ2 NSs. (**E**) Schematic comparing molecular packing in NPs and NSs. (**F** and **G**) MD simulation snapshots of TNQ2 aggregates and TNQ2-P123 aggregates in water. (**H**) Radius of gyration for TNQ2 aggregates and TNQ2-P123 aggregates. (**I** to **K**) Time-dependent interaction energy plots: overall system (I), TNQ2 aggregates (J), and TNQ2-P123 aggregates (K). (**L**) Dihedral angle distributions between phenyl rings and pyrene planes for the innermost molecules in TNQ2 aggregates and TNQ2-P123 aggregates in water. (**M**) Packing diagrams illustrating *J*-aggregate formation among TNQ2 molecules in TNQ2-P123 aggregates. (**N**) MD simulation of the time-dependent slip angle profile of TNQ2 molecules in TNQ2-P123 aggregates.

Subsequently, TNQ2 molecules were assembled into nanomaterials. In the absence of amphiphilic surfactants, TNQ2 molecules dissolved in THF rapidly precipitated upon solvent evaporation, forming monodispersed spherical nanoparticles (NPs) with an average size of 73.1 nm (Fig. 3A and fig. S13). When amphiphilic block polymers were introduced, TNQ2 molecules assembled into well-defined 2D TNQ2 NSs: DSPE-PEG induced NSs with a length (*L*) of 3.5 μm and width (*W*) of 2.7 μm [aspect ratio (AR) = 1.3], DSPE-PEG-COOH yielded NSs (*L*: 1.9 μm, *W*: 1.0 μm, and AR = 1.9), Pluronic F127 produced NSs (*L*: 1.8 μm, *W*: 1.4 μm, and AR = 1.3), and block polymer (Pluronic P123) generated the smallest NSs (*L*: 0.16 μm, *W*: 0.11 μm, and AR = 1.5). The atomic force microscopy (AFM) image revealed that TNQ2 NSs formed with P123 had an average thickness of only 14.3 nm (Fig. 3B), making them more suitable for in vivo theranostic applications, particularly for traversing the BBB compared to other variants. Unless otherwise specified, all subsequent references to TNQ2 NSs were the NSs assembled with P123. As shown in Fig. 3 (C and D), the maximum absorption wavelength of TNQ2 molecules (545 nm) red-shifted to 595 nm for TNQ2 NPs and 725 nm for TNQ2 NSs, whereas their emission peaks at around 1000 nm remained largely unchanged. Notably, the emission intensity of NSs increased substantially than NPs and monomer, collectively indicating the formation of *J*-aggregates in TNQ2 NSs (Fig. 3E). TNQ2 NSs exhibited excellent colloidal stability, with minimal size variation over 5 days in serum and common media, and a zeta potential of ~−19.3 mV, comparable to that of Ang-TNQ2 NSs (−21.3 mV), collectively supporting their stability under physiological conditions (fig. S14). X-ray diffraction (XRD) analysis further revealed amorphous aggregation in raw TNQ2 powder and TNQ2 NPs, whereas distinct crystal peaks in TNQ2 NSs suggested oriented molecular stacking along certain directions (fig. S15). In addition, the concentration-dependent absorption spectra (fig. S16) showed that the maximum absorption band of TNQ2 remarkably red-shifted at high concentrations or in the solid film state, possibly resulting from the exciton coupling in *J*-aggregates with close packing. Moreover, the absolute fluorescence quantum yields (Φ) and fluorescence lifetimes (τ) of the solution, NPs, and NSs of TNQ2 were measured, based on which the radiative transition rates (*k*_r_) and nonradiative transition rates (*k*_nr_) were calculated. As shown in fig. S17 and table S3, the Φ values of NPs and NSs (0.3%) were substantially higher than that of the solution state (0.02%), further confirming the AIE effect of TNQ2. The remarkably shortened τ from NPs to NSs accompanied by an obviously increased *k*_r_ value were consistent with the characteristic features of *J*-aggregates. The photophysical characterization results collectively suggested the *J*-aggregate formation possibility in TNQ2 NSs.

To gain deeper insight into the aggregation characteristics and intermolecular interactions, molecular dynamics (MD) simulations were carried out for the aggregates of TNQ2 in water in the absence or presence of P123 using GROMACS software with the general assisted model building with energy refinement (AMBER) force field (*46*). The molecular geometries of TNQ2 and P123 were first optimized using DFT. Subsequently, aggregates comprising 10 TNQ2 molecules alone and aggregates comprising 10 TNQ2 and ten P123 molecules were constructed, respectively, and solvated in a cubic water box with an edge length of 10 nm for MD simulations. Figure 3 (F and G) and movies S1 and S2 showed the spatial arrangement and optimized structures of TNQ2 aggregates and TNQ2-P123 aggregates. We speculated that TNQ2 are inclined to form aggregates in a disordered manner in the absence of surfactants. For TNQ2-P123 aggregates, the hydrophobic nature of TNQ2 molecules make them tend to locate inside the polymers with the surfactant on the aggregate surfaces, and the intermolecular interactions of TNQ2 may change accordingly to induce the formation of relatively more ordered *J*-aggregates. The intermolecular interaction energy analysis between TNQ2 and P123 in the simulation trajectory of TNQ2-P123 aggregates further confirmed that P123 mainly affected TNQ2 molecules via the hydrophobic van der Waals (VDW) forces (table S4). To assess the compactness, the gyration radius of the aggregates was calculated by analyzing the simulation trajectory. As depicted in Fig. 3H, the gyration radius of TNQ2-P123 aggregates was smaller than that of the TNQ2 aggregates. Besides, the absolute intermolecular interaction energy among TNQ2 molecules became higher in the presence of P123 in the aggregate state (Fig. 3I). All these results suggested that TNQ2 molecules stack in a more compact way in the NSs with the assistance of polymer surfactants. The proportion of Coulomb short range and reciprocal interactions between TNQ2 molecules increased in TNQ2-P123 aggregates, but the hydrophobic VDW interactions still serve as the main driving force for molecular aggregation (Fig. 3, J and K, and tables S5 and S6). The enhanced aggregation ability of TNQ2 in the presence of P123 is consistent with the higher PL intensity of TNQ2 NSs than that of TNQ2 NPs as the RIM effect can be activated more effectively by the tighter stacking of TNQ2-P123 aggregates.

To investigate the molecular motions of TNQ2 under different aggregate conditions, we calculated the dihedral angle between the phenyl and pyrene moiety and the dihedral angle between the phenyl and the malononitrile-containing cyclohexadiene plane of the innermost TNQ2 molecule in the absence and presence of P123. As depicted in Fig. 3L and fig. S18, both dihedral angles increased in TNQ2-P123 aggregates. The relatively more twisted conformation of TNQ2 molecule in the presence of P123 might also contribute to the stronger PL intensity of NS aggregates. Further analysis of the molecular packing among TNQ2 molecules in different aggregates revealed that there existed multiple intermolecular C─H···π interactions in both aggregates and C─N···π interactions in TNQ2 aggregates to facilitate the RIM processes (figs. S19 and S20). It is notable that characteristic *J*-stacking was observed in the simulated P123-decorated TNQ2 aggregates. As depicted in Fig. 3M, the slip angle between the pyrene planes of two adjacent TNQ2 molecules was measured to be 33.6°, which is smaller than the critical value of 54.7°, demonstrating the formation of *J*-aggregates (*35*). Similarly, the slip angle between the cyclohexadiene moiety and the pyrene moiety and the slip angle between the phenyl rings of two neighboring TNQ2 molecules was calculated to be 45.7° and 45.5°, which further confirmed the occurrence of *J*-aggregation. In some circumstances, *H*-aggregates was also detected in TNQ2-P123 aggregates (fig. S21), which is consistent with the increased *k*_nr_ value of TNQ2 NPs (table S3), but *J*-aggregates still serve as the main packing mode. As evidenced by the MD-derived slip angle distribution (Fig. 3N), TNQ2 in P123-decorated aggregates predominantly adopts slip angles of 10° to 40° with an average of 20.68°, which clearly verified our proposed speculation. The radial distribution function (fig. S22) revealed a preferred intermolecular distance of 3.78 Å between TNQ2 molecules in TNQ2-P123 aggregates, indicating the presence of stable π-π stacking and other interactions that facilitate highly ordered molecular packing. Together, the formation of *J*-aggregates among TNQ2 after adding P123 may play a key role in the photophysical properties.

### NIR photoenergy transformation of NSs

**Fig. 4.**
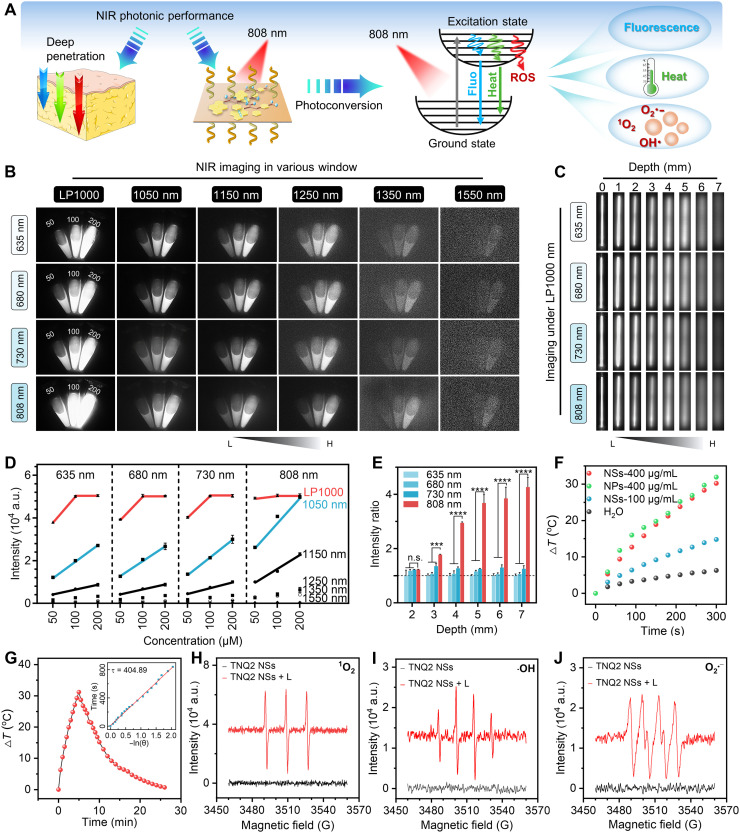
Multimodal photoenergy utilization of TNQ2 NSs. (**A**) Schematic of NIR fluorescence, photothermal, and photodynamic effects generated by TNQ2 NSs under 808-nm irradiation. (**B**) NIR fluorescence images of TNQ2 NS aqueous solutions at various concentrations (μM) under different excitation wavelengths (635, 680, 730, and 808 nm) and emission channels (LP1000, 1050, 1150, 1250, 1350, and 1550 nm). (**C**) NIR fluorescence images (LP1000 channel) of capillary-encapsulated TNQ2 NSs under various excitation wavelengths through different thicknesses of tissue samples. (**D** and **E**) Quantitative NIR fluorescence intensity (*n* = 3) corresponding to (B) and (C), respectively. (**F**) Photothermal curves of TNQ2 NSs and TNQ2 NPs at specified concentrations under 808-nm irradiation (1 W/cm^2^). (**G**) Corresponding cooling curve of TNQ2 NSs after irradiation. (**H** to **J**) ESR spectra demonstrating ROS generation (^1^O_2_, •OH, and O_2_^•−^) by TNQ2 NSs under 808-nm irradiation. All data were presented as means ± SD. n.s., not significant; ***P < 0.001; ****P < 0.0001. All statistical data were compared using one-way ANOVA.

The NIR photoenergy transformation of TNQ2 NSs involves three primary modes: fluorescence, photothermal effects, and photoinduced ROS generation through radiative and nonradiative transition pathways (Fig. 4A). Initially, the NIR fluorescence imaging capability of TNQ2 NSs was evaluated using an NIR imaging system, revealing strong fluorescence across multiple excitation (635, 680, 730, or 808 nm) and emission (LP1000, 1050, 1150, 1250, 1350, and 1550 nm) channels (Fig. 4B). Notably, 808-nm excitation efficiently activated NIR emission even through 7 mm of tissue depth (Fig. 4C), as confirmed by fluorescence intensity quantification (Fig. 4, D and E). TNQ2 NSs retained 99.6% of their initial absorbance after irradiation, far exceeding the 7.4% retained by indocyanine green (ICG), which demonstrates the superior photostability (fig. S23). In addition to fluorescence, TNQ2 NSs also exhibited pronounced photothermal and photodynamic effects. Under 808-nm irradiation, TNQ2 NSs exhibited a concentration-dependent temperature change, showing a 30.2°C temperature increase at 400 μg/mL. The temperature rise of TNQ2 NPs was slightly higher than NSs at the same concentration (Fig. 4F), possibly due to the relatively more disordered packing manners of TNQ2 within NPs. The photothermal conversion efficiency of TNQ2 NSs was determined to be 20.4% (Fig. 4G), and the photothermal performance retained stable over five consecutive irradiation cycles (fig. S24). To further evaluate their photodynamic potential, we examined the ROS generation capability of both TNQ2 NPs and NSs using the fluorescent ROS probe 2,7-dichlorodihydrofluorescein diacetate (DCFH-DA). The results (fig. S25) confirmed that TNQ2 NSs produce higher ROS levels than TNQ2 NPs upon 808-nm irradiation. Subsequently, the major ROS species of TNQ2 NSs generated through the photodynamic effect, including singlet oxygen (^1^O_2_), hydroxyl radical (•OH), and superoxide radical (O_2_^•−^), were assessed by electron spin resonance (ESR). Substantially enhanced characteristic peaks were observed (Fig. 4, H to J), confirming the strong ROS generation under 808-nm irradiation. Last, the ROS of TNQ2 NSs was quantitatively evaluated using methylene blue (MB) as a standard probe (Φ_Δ_ = 0.53). The calculated Φ_Δ_ values for TNQ2 NSs reached 0.61 under 635-nm irradiation, representing high PDT potential (fig. S26). These results demonstrate the superior NIR photoresponsive theranostic potential of TNQ2 NSs, supporting their application in subsequent surgery navigation and postoperative phototherapy for glioma.

### In vitro phototherapy and imaging-guided surgery

**Fig. 5.**
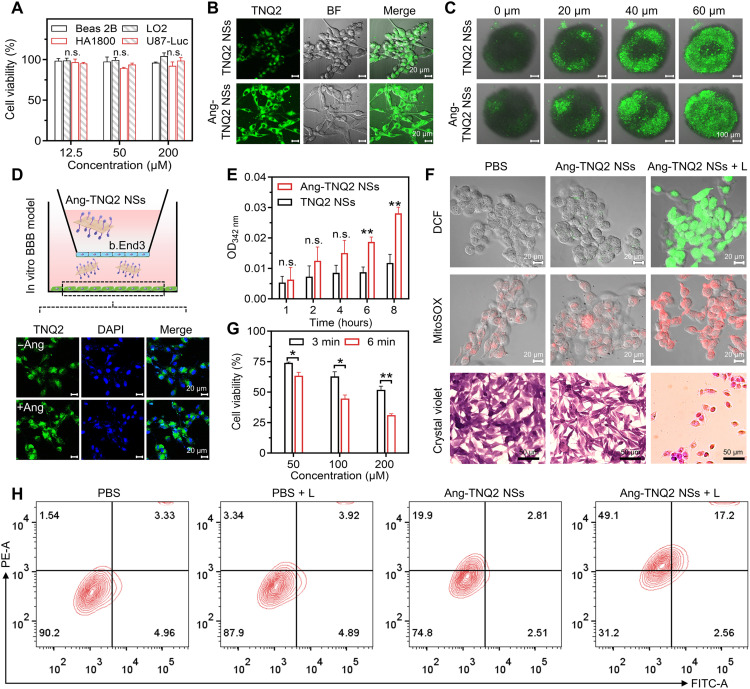
In vitro phototherapy effects of Ang-TNQ2 NSs. (**A**) Cytotoxicity of Ang-TNQ2 NSs in different cell lines (*n* = 3). (**B**) Confocal images comparing the cellular uptake of TNQ2 NSs and Ang-TNQ2 NSs in U87 cells. (**C**) Confocal images for the 3D tumor spheroid penetration of two NSs. (**D**) Schematic of Transwell assay and the corresponding confocal image showing TNQ2 NSs and Ang-TNQ2 NSs traversing the BBB. (**E**) Absorption quantification of penetrated NSs (*n* = 4). OD, optical density. (**F**) Confocal images of ROS detected by the green DCFH-DA probe and mitochondrial superoxide tested by the red Mito-SOX probe in U87 cells. The third row shows the corresponding crystal violet staining images in U87 cells. (**G**) Cell viability of U87 cells treated with Ang-TNQ2 NSs at different concentrations under irradiation for 3 or 6 min (*n* = 3). (**H**) Flow cytometry analysis of U87 cells treated with PBS, PBS with 808-nm irradiation (L) for 9 min, Ang-TNQ2 NSs (200 μM), and Ang-TNQ2 NSs (200 μM) + L (9 min). Unless specified, all cells were U87-luciferase cells (termed as U87), all irradiations were 808-nm irradiation (1.0 W/cm^2^), and the concentrations of NSs were 100 μM. Data are presented as means ± SEM. Statistical analysis was performed using one-way ANOVA (A) and a two-tailed Student’s *t* test [(E) and (G)]. *P < 0.05; **P < 0.01; n.s., not significant.

To study the targeted phototherapy in glioma cells, TNQ2 NSs were modified with brain-targeted Ang-2 peptide (Ang-TNQ2 NSs). The transmission electron microscopy (TEM) image of Ang-TNQ2 NSs clearly demonstrated the sheetlike morphology (fig. S27). First, the toxicity of Ang-TNQ2 NSs was assessed in three normal cell lines (Beas 2B, LO2, and HA1800) and the U87-luciferase cell line (U87-Luc), showing negligible dark cytotoxicity (Fig. 5A). Then, confocal imaging in U87 cells (Fig. 5B) and 3D tumor spheroids (Fig. 5C) demonstrated enhanced uptake and tumor penetration of Ang-TNQ2 NSs. Transwell assays further demonstrated higher (2.4-fold) time-dependent BBB penetration increasement of Ang-TNQ2 NSs than nontargeted group (Fig. 5, D and E).

Subsequently, the phototherapy efficiency was tested in U87 and EC109 cells. The Ang-TNQ2 NSs with the irradiation group showed substantially elevated ROS (DCFH-DA probe) and mitochondrial superoxide (Mito-SOX probe) in two cell lines compared to phosphate-buffered saline (PBS) and Ang-TNQ2 NS groups (Fig. 5F and figs. S28 and S29). Dihydroethidium (DHE) staining further confirmed the intracellular generation of superoxide anions in U87-Luc cells by Ang-TNQ2 NSs upon 808-nm irradiation, as evidenced by distinct red fluorescence (fig. S30). Crystal violet staining revealed decreased cells and pronounced cell shrinkage in U87 cells treated with Ang-TNQ2 NSs with irradiation than the other two groups. Phototoxicity assays demonstrated concentration-dependent and time-dependent cell damage, i.e., U87 cell viability decreased to 51.8 and 31.1% by 200 μM Ang-TNQ2 NSs with 3 and 6 min of irradiation (Fig. 5G), as well as 31.7% of EC109 cells by cyclic Arg-Gly-Asp (cRGD)–modified NSs (cRGD-TNQ2 NSs) with 808-nm irradiation for 6 min (fig. S31). In flow cytometry analysis, compared to 90.2% live cell of the PBS group, 87.9% in light-only group, and 74.8% in Ang-TNQ2 NS groups, the Ang-TNQ2 NSs with the irradiation group decreased to 31.2% (Fig. 5H), demonstrating exceptional in vitro targeted phototherapeutic efficacy of Ang-TNQ2 NSs.

**Fig. 6.**
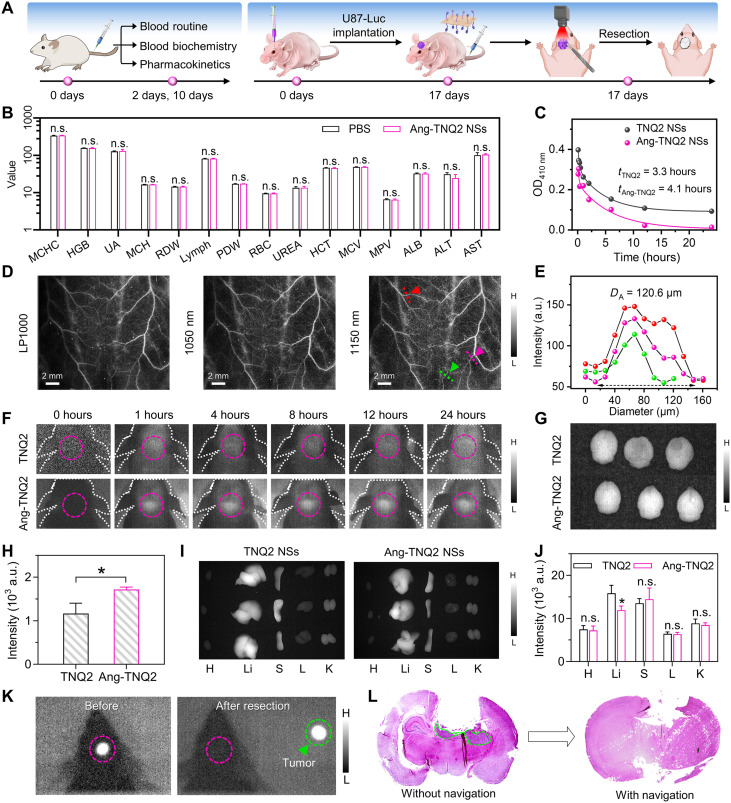
NIR fluorescence imaging-guided surgical navigation. (**A**) Schematic of in vivo biosafety assessment and NIR imaging-guided surgical navigation. (**B**) Blood routine and blood biochemical parameters comparison between normal mice injected with PBS or Ang-TNQ2 NSs (*n* = 3). (**C**) Pharmacokinetic profiles of TNQ2 NSs and Ang-TNQ2 NSs in mice. (**D** and **E**) NIR imaging through long-pass 1000-nm filter (LP1000), 1050, and 1150 nm filters of vessel (D) and quantifiable vessel diameter analysis (E) in mice injected with Ang-TNQ2 NSs. (**F**) In vivo NIR fluorescence imaging of orthotopic glioma-bearing mice over time after administration of TNQ2 NSs and Ang-TNQ2 NSs. (**G** and **H**) Corresponding NIR images (G) and quantification (H) of ex vivo brains from two groups (*n* = 3). (**I**) NIR images of ex organs of mice (*n* = 3) at 24 hours postinjection of TNQ2 NSs and Ang-TNQ NSs. The heart, liver, spleen, lung, and kidney were abbreviated as H, Li, S, L, and K. (**J**) Biodistribution in major organs of mice (*n* = 3). (**K**) Real-time NIR fluorescence (1250 nm) imaging-guided glioma resection. (**L**) H&E-stained images of brain slices after resection with or without navigation guidance. Unless otherwise stated, all dosages were 10 mg/kg. Data are presented as means ± SD. Statistical analyses were performed using a two-tailed Student’s *t* test. *P < 0.05; n.s., not significant.

Next, we evaluated the real-time NIR intraoperative navigation efficiency of Ang-TNQ2 NSs in an orthotopic glioblastoma model (Fig. 6A). The biosafety of Ang-TNQ2 NSs was first confirmed through hemolytic tests, blood routine, and blood biochemical analyses, demonstrating excellent biocompatibility (Fig. 6B and fig. S32). Pharmacokinetic studies revealed circulation half-lives of 3.3 hours for TNQ2 NSs and 4.1 hours for Ang-TNQ2 NSs (Fig. 6C), providing a favorable foundation for precise surgical navigation. High-sensitivity NIR imaging clearly delineated the vascular network in mice injected with Ang-TNQ2 NSs, achieving an average vessel resolution of 120.6 μm (Fig. 6, D and E). Real-time NIR fluorescence tracking in the brain confirmed the BBB-penetrating ability and glioma-targeting specificity of Ang-TNQ2 NSs, with peak tumor accumulation occurring at 8 hours postinjection (Fig. 6F). The NIR fluorescence images and quantification of ex vivo brain confirmed that Ang-2 modification substantially enhanced BBB penetration (Fig. 6, G and H). Biodistribution studies revealed that both nanomaterials primarily accumulated in the liver and spleen due to the reticuloendothelial system (Fig. 6I). Further intensity quantification of Ang-TNQ2 NS distribution also showed predominant accumulation in the liver and spleen (Fig. 6J). Under the guidance of high-sensitivity real-time NIR imaging at 1250 nm, Ang-TNQ2 NSs achieved precise glioma resection (Fig. 6K), as evidenced by reduced tumor residue in hematoxylin and eosin (H&E) staining brain sections (Fig. 6L). Collectively, these results underscore the superior in vivo imaging performance of Ang-TNQ2 NSs, enabling real-time and precise glioma resection.

### In vivo phototherapy study

**Fig. 7.**
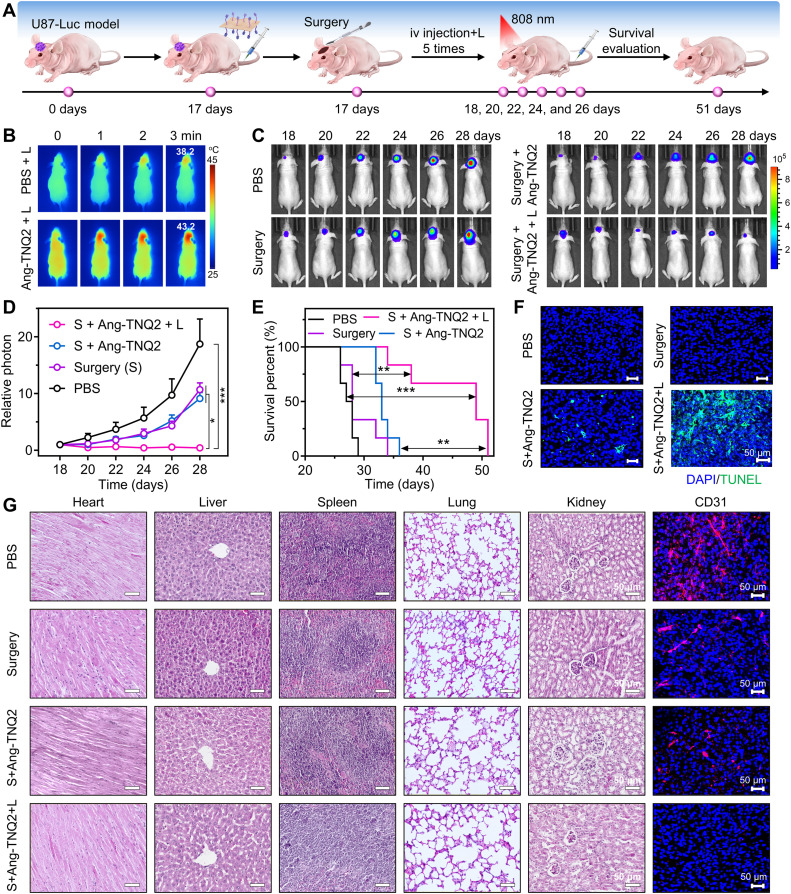
In vivo phototherapy of Ang-TNQ2 NSs in postoperative mouse models. (**A**) Experimental design and timeline for phototherapy evaluation. iv, intravenous. (**B**) In vivo photothermal images of mice treated with PBS and Ang-TNQ2 NSs under 808-nm irradiation. (**C** and **D**) In vivo bioluminescence images (C) and the corresponding intensity quantification (D) of tumor in U87 glioma-bearing mice with various treatments (*n* = 5). Surgery is abbreviated as S. (**E**) Survival curves of mice receiving the indicated treatments (*n* = 6). Differences between groups were analyzed by the log-rank test. (**F**) TUNEL staining of mice with the indicated treatments. (**G**) H&E staining of major organs and immunofluorescence staining of brain sections. Unless otherwise stated, all injected dosages were 10 mg/kg and all irradiations (L) were 808 nm (0.5 W/cm^2^) for 5 min. Data in (D) are presented as means ± SEM. Statistical analysis in (D) was performed using one-way ANOVA. *P < 0.05; **P < 0.01; ***P < 0.001.

Last, to further eliminate residual tumors postresection, NIR phototherapy was performed on postoperative mouse models following the experimental workflow illustrated in Fig. 7A. In vivo photothermal imaging showed that the brains of glioma-bearing mice injected with Ang-TNQ2 NSs reached 43.2°C under irradiation, demonstrating efficient photothermal conversion. This temperature was substantially higher than the 38.2°C observed in the PBS-injected control group (Fig. 7B). Furthermore, the ex vivo brain tissues from the Ang-TNQ2 NS group showed markedly higher DCFH-DA fluorescence upon 808-nm irradiation, indicating strong in vivo ROS generation. By contrast, only negligible fluorescence was detected in the nonirradiated Ang-TNQ2 NS group and the PBS control group (fig. S33). Upon 808-nm irradiation, the combined photothermal and ROS effects triggered efficient residual tumor cell ablation. Bioluminescence imaging and corresponding intensity quantification of tumor validated the effective phototherapeutic ablation by Ang-TNQ2 NSs (Fig. 7, C and D). As showed in Fig. 7E, Ang-TNQ2 NSs with irradiation effectively suppressed residual tumor recurrence, substantially prolonging survival to 51 days, compared to 29 days (PBS group), 34 days (surgery alone group), and 36 days (surgery with Ang-TNQ2 NS group). Terminal deoxynucleotidyl transferase–mediated deoxyuridine triphosphate nick end labeling (TUNEL) and blood vessel endothelial cell adhesion molecule–1 (CD31) analyses revealed that Ang-TNQ2 NSs induced the highest levels of tumor cell death and efficient vascular damage under 808-nm irradiation (Fig. 7, F and G). Meanwhile, H&E staining results showed no notable tissue damage in the heart, liver, spleen, lung, and kidney, confirming negligible side effects and high biocompatibility. Furthermore, long-term toxicity was assessed in healthy mice via continuous intravenous injection of Ang-TNQ2 NSs (10 mg/kg) for 1 month (15 injections). H&E staining analysis similarly revealed no observable toxicity compared to the PBS control group (fig. S34), which further supports the favorable biosafety profile of Ang-TNQ2 NSs. The potent phototherapeutic efficacy and versatility of TNQ2 NSs were further validated with cRGD-modified TNQ2 NSs (cRGD-TNQ2 NSs) in a nonsurgical subcutaneous EC109 tumor model. Tumor images, tumor size tracking, and weight measurements (fig. S35, A to C) demonstrated excellent tumor ablation, supported by efficient apoptosis generation (fig. S35D). Collectively, these in vivo results highlight the strong targeted tumor ablation capability of TNQ2 NSs.

## DISCUSSION

In summary, this study offers a strategy for addressing molecular and nanoengineering challenges related to NIR photoregulation. The click-to-twist feature of the synthetic strategy yields D-π-A–type AIE systems with highly twisted conformations and ultralarge Stokes shifts of up to 445 nm. It tackles the long-standing challenges in designing and synthesizing simple NIR-II fluorophores with large Stokes shifts based on the facile one-step click reactions of TCNQ derivatives and pyrene-containing asymmetric internal alkyne. With the assistance of P123, TNQ2 molecules were self-assembled into ultrathin 2D rectangular NSs with a length/width of 160 nm by 110 nm, which are the smallest 2D NIR-II AIE NSs reported to date. *J*-aggregation in TNQ2 NSs exhibited a substantial red shift in absorption (725 nm) and remarkably enhanced NIR-II fluorescence intensity. This work represents the achievement of the largest Stokes shift spanning from visible to NIR-II regions, enabling precise surgical navigation for glioblastoma resection after efficiently traversing the BBB. Furthermore, by strategically balancing radiative and nonradiative processes via molecular structure design, the fluorescence, photothermal, and photodynamic performances were optimized to achieve exceptional postoperative glioma clearance. The tumor ablation capability of this NIR theranostic agent has also been validated in other tumor models. The exceptional flexibility, simplicity, and high therapeutic efficiency of our 2D NIR-II nanosystem with an ultralarge Stokes shift potentially transform the current paradigm of NIR-II theranostic tool development and landscape for clinical applications.

## MATERIALS AND METHODS

### General information

All chemicals and reagents were purchased from commercial sources and used as received without further purification. ^1^H NMR and ^13^C NMR spectra were performed on 400-, 500-, and 600-MHz NMR spectrometers (Bruker AVANCE). Chemical shifts were calibrated using CDCl_3_ [^1^H NMR: δ 7.26 parts per million (ppm); ^13^C NMR: δ 77.16 ppm] as an internal reference. HRMS analyses were performed on an integrated liquid chromatography–tandem mass spectrometry (LC-MS/MS) system, comprising a Thermo Fisher Scientific Ultimate 3000 RSLC HPLC unit and a Q Exactive Orbitrap mass spectrometer, and HRMS data were reported with ion mass/charge ratios (*m*/*z*) as values in unified atomic mass units. Ultraviolet-Visible-NIR absorption spectra were measured on a PerkinElmer Lambda 950 spectrophotometer. The fluorescence spectra were tested by FluoroLog-3 (HORIBA) fluorescence spectrophotometer (treatment method of connecting line across missing data was used to remove the overtone bands in emission spectra with an excitation wavelength of 635 nm). The solvatochromic PL measurements of TNQ2 were recorded on FluoroMax^+^ fluorescence spectrophotometer (HORIBA). Fluorescence lifetimes were performed on FLS980 fluorescence spectrometers of Edinburgh. The absolute fluorescence quantum yields were measured by Hamamatsu C13534-31. The animal assays were administrated following the Guide for the Care and Use of Laboratory Animals and approved by the Medical and Scientific Research Ethics Committee of Henan University School of Medicine (approval ID: HUSOM-2018-355).

### Preparation of Ang-TNQ2 nanomaterials

TNQ2 (0.1 mg) was dissolved in THF (100 μL), and the solution was added to 2 mL of an aqueous Pluronic P123 solution (1.0 mg/mL). The mixture was stirred gently (500 rpm) at room temperature for 12 hours to allow self-assembly. The resulting suspension was centrifuged (15,000 rpm, 20 min) and washed twice with ultrapure water to remove free polymer, yielding purified TNQ2 NSs. For peptide conjugation, the Ang-SH peptide (1.0 mg) was added to 1.0 mL of the TNQ2 NS aqueous dispersion (1.0 mg/mL). The mixture was sonicated for 5 min and then stirred continuously for 12 hours at room temperature. The final Ang-TNQ2 NSs were collected by centrifugation (15,000 rpm, 20 min) and washed twice with deionized water. Other TNQ2 nanomaterials were prepared similarly by substituting DSPE-PEG (1.0 mg/mL), F127 (1.0 mg/mL), or DSPE-PEG-COOH (1.0 mg/mL) for P123 in the initial self-assembly step.

### Cell culture

The U87-Luc cell was bought from Shanghai Model Organisms Center Inc. (catalog number: NM-N01). The EC109 cell was obtained from the National Collection of Authenticated Cell Cultures (China, catalog number: SCSP-5455). All experiments were performed using luciferase-expressing U87 cells (U87-Luc) unless otherwise specified. U87-Luc cells were cultured in Dulbecco’s modified Eagle’s medium (DMEM) supplemented with 10% fetal bovine serum (FBS), 1% penicillin-streptomycin, and 1% nonessential amino acids. EC109 cells were maintained in RPMI 1640 medium containing 10% FBS, 1% penicillin-streptomycin, and 1% nonessential amino acids. All cell lines were incubated at 37°C in a humidified atmosphere of 5% CO_2_.

### Intracellular ROS detection

To evaluate in vitro ROS generation, U87 cells were seeded in glass-bottom dishes (3 × 10^4^ cells per well in 300 μL of medium) and cultured overnight. Subsequently, the medium was replaced with fresh complete medium containing Ang-TNQ2 NSs (100 μM) and the cells were incubated for 8 hours, followed by irradiation with an 808-nm laser (1.0 W/cm^2^) for 9 min. For ROS detection, the cells were then stained with DCFH-DA (10 μM) for total ROS and MitoSOX Red (5 μM) for mitochondrial superoxide at 37°C for 15 min, and fluorescence imaging was performed using a confocal laser scanning microscope with excitation at 488 nm (for DCFH-DA) and 514 nm (for MitoSOX).

### 3D tumorsphere penetration evaluation

The penetration capability of NPs was assessed using an in vitro 3D tumor spheroid model. Tumor spheroids were generated by seeding U87-Luc cells (1 × 10^4^ cells per well) in ultralow attachment 96-well plates and culturing for 48 hours to allow spheroid formation. For penetration studies, mature spheroids were incubated with either Ang-TNQ2 NSs or TNQ2 NSs (100 μM) for 8 hours and then washed three times with PBS to remove unbound NSs. The penetration depth of TNQ2 NSs into the spheroids was visualized using confocal laser scanning microscopy (Zeiss LSM 880; excitation wavelength: 488 nm) via *z*-stack imaging.

### In vitro BBB penetration evaluation

An in vitro BBB model was established by seeding b.End3 cells (National Collection of Authenticated Cell Cultures, China; catalog number: SCSP-5267) at a density of 5 × 10^4^ cells per well onto Transwell inserts and cultured for 48 hours. The model integrity was verified by measuring transendothelial electrical resistance (TEER) using an epithelial voltohmmeter; models exhibiting TEER values exceeding 200 Ω·cm^2^ were used for subsequent experiments. For the penetration assay, U87-Luc cells (1 × 10^5^ cells per well) were seeded in the lower chambers after 24 hours of b.End3 cell culture, followed by 24 hours of coculture after which the medium was replaced with fresh medium containing TNQ2 NSs or Ang-TNQ2 NSs (100 μM). Samples from the lower chamber were collected at predetermined time points (1, 2, 4, 6, and 8 hours) for spectrophotometric analysis at 342 nm, whereas penetration efficiency was further assessed indirectly by evaluating NSs uptake in U87-Luc cells via confocal laser scanning microscopy.

### In vitro phototherapy assessment

The synergistic therapeutic effect of Ang-TNQ2 NSs was assessed in U87 glioma cells via CCK-8 viability assay (*n* = 3). Briefly, cells (5 × 10^3^ cells per well) were seeded in 96-well plates and allowed to adhere overnight. The cells were then incubated with various concentrations (50, 100, and 200 μM) of Ang-TNQ2 NSs or PBS for 8 hours, after which the medium was replaced with fresh medium. Following two washes with PBS, cells were irradiated with an 808-nm laser (1.0 W/cm^2^) for varying durations. After overnight incubation, cell viability was measured by adding 10 μL of CCK-8 reagent to each well and incubating for 1 hour, followed by absorbance measurement at 450 nm using a microplate reader (BioTek Synergy H1). The same procedure was subsequently applied to EC109 cells for further validation.

### In vivo pharmacokinetics study

The blood circulation profile of Ang-TNQ2 NSs was evaluated in healthy BALB/c mice. Mice were intravenously administered Ang-TNQ2 NSs or TNQ2 NSs at a dosage of 10 mg/kg. Blood samples were collected at predetermined time points and centrifuged at 3000 rpm for 15 min at 4°C. The supernatants were transferred to a 96-well plate, and the absorbance at 410 nm was measured using a microplate reader.

### Establishment of orthotopic glioma-bearing mouse models

Female BALB/c nude mice (6 to 8 weeks old) were purchased from SiPeiFu (SPF) Biotechnology Co. Ltd. (Beijing, China). To establish the orthotopic glioma model, U87-Luc cells (2 × 10^5^ cells in 5 μL of NaCl) were stereotactically injected into the left striatum (depth: 3 mm) of anesthetized mice. Tumor growth was monitored using an IVIS Lumina III imaging system (PerkinElmer) following intraperitoneal injection of d-luciferin potassium (75 mg/kg).

### NIR fluorescence-guided surgical navigation

On day 17 postimplantation, orthotopic glioma-bearing mice were intravenously administered Ang-TNQ2 NSs (10 mg/kg) and subjected to tumor resection under real-time NIR-II fluorescence guidance. At 8 hours postinjection, intraoperative imaging was performed using an NIR-II fluorescence system (Ex: 808 nm; Em: 1250 nm) to delineate tumor margins. Following resection, the excised brain tissues were fixed in 4% paraformaldehyde for 24 hours and subsequently processed for H&E staining to confirm resection completeness and evaluate tumor and peritumoral morphology.

### Postoperative phototherapy in surgical model mice

To evaluate the ablation efficacy of Ang-TNQ2 NSs on residual tumor, mice were randomly divided into four groups (*n* = 5 per group): surgery-only, surgery + Ang-TNQ2 NSs, surgery + Ang-TNQ2 NSs + L (irradiation), and nonsurgical PBS control. After resection, these mice were intravenously injected with either PBS or Ang-TNQ2 NSs (dosage: 10 mg/kg), followed by 808-nm irradiation (0.5 W/cm^2^) for 5 min. The injection/irradiation cycles were repeated five additional times at 2-day intervals. Throughout the therapy process, tumor growth was monitored using the IVIS Lumina III imaging system in the whole therapy process and survival of mice was simultaneously recorded. After finishing therapy, the major organs (brain, heart, liver, spleen, lungs, and kidneys) of mice were harvested for histopathological analysis. Tissues were fixed in 4% paraformaldehyde, paraffin embedded, and sectioned for H&E staining to evaluate therapy efficiency and biosafety.

### In vivo phototherapy assessment in subcutaneous EC109 tumor models

Subcutaneous EC109 tumor models were established by injecting EC109 cells (3 × 10^6^ in 50 μL of PBS) into the thigh region of nude mice. Mice were randomly assigned to two treatment groups (*n* = 3 per group): PBS (control) and cRGD-TNQ2 NSs (10 mg/kg). Mice were irradiated at the tumor site with an 808-nm laser (0.5 W/cm^2^) for 5 min following administration. Body weight and tumor volume were recorded every 2 days throughout the study. After treatment, the mice were euthanized and the tumor was prepared into section for TUNEL microscopy imaging.

### Tissue section staining and observation

Tissue sections of main organs (heart, liver, spleen, lung, and kidney) were baked at 65°C for 2 hours and dewaxed in xylene and gradient ethanol (100, 90, 80, and 75%). Sections were stained with hematoxylin (5 min), rinsed, counterstained with eosin (3 min), dehydrated through graded ethanol (75, 80, 90, and 100% ethanol), and mounted with xylene-neutral resin (1:1, v/v). For brain sections, after dewaxing, antigen repair was performed using microwave treatment (medium-high heat, 2 min) followed by 10 cooling cycles (20-s heating/2-min cooling). Sections were incubated with CD31 primary antibody (Abcam, 1:100) at 4°C overnight, followed by incubated by secondary antibody (UElandy, 1:1000, 1 hour) and 4′,6-diamidino-2-phenylindole (DAPI) (10 min). Slides were mounted with antifade medium. Apoptotic signals in brain tumors were detected using a TUNEL assay kit (Beyotime Biotechnology).

### Hemolysis test

Whole blood (0.2 ml) was collected from Balb/c mice and divided into six groups. The erythrocyte pellet was centrifuged at 3000 rpm for 2 min and washed repeatedly with PBS until the supernatant was colorless. These erythrocyte pellet respectively mixed with 1.0 mL of the following solutions: ultrapure water (positive control, 100% hemolysis), PBS (negative control, 0% hemolysis), or TNQ2 NSs in PBS at four concentrations (12.5, 25, 50, and 200 μM). All mixtures were incubated at 37°C for 2 hours. Subsequently, the samples were centrifuged at 15,000 rpm for 20 min. The supernatant was collected, and its absorbance at 541 nm was measured using a microplate reader in a 96-well plate.

### Blood routine and blood biochemical assessments

BALB/c mice were randomly divided into two groups: the PBS-treated control group and the Ang-TNQ2 NSs-treated group. Mice received a single intravenous injection of PBS or Ang-TNQ2 NSs (dosage: 10 mg/kg). At 10 days postinjection, the blood samples were collected from all mice. A portion of blood was used for blood routine analysis. The upper serum in another blood samples was harvested after centrifugation at 3000 rpm and 4°C for 15 min. They were then subjected to biochemical analysis to assess liver and kidney function markers including alanine aminotransferase (ALT) and aspartate aminotransferase (AST) to provide detailed biosafety profiles beyond hemolysis.

### Long-term biosafety evaluation

Balb/c mice were intravenously injected with either PBS (control) or Ang-TNQ2 NSs (10 mg/kg) via the tail vein every other day for 30 days (15 doses in total). After the treatment regimen, the major organs (heart, liver, spleen, lung, and kidney) of mice were harvested. The organs were then fixed, sectioned, and subjected to H&E staining for histopathological analysis to evaluate the long-term biosafety of the nanomaterials.

### Statistical analysis

All quantitative data are expressed as means ± SD or SEM as specified in figure legends, with a minimum of three independent biological replicates. Intergroup comparisons between two datasets were performed using unpaired two-tailed Student’s *t* test, whereas differences among multiple groups were assessed by one-way analysis of variance (ANOVA). Survival analysis was conducted using the log-rank (Mantel-Cox) test. Statistical significance thresholds were defined as follows: *P < 0.05, **P < 0.01, ***P < 0.001, ****P < 0.0001, n.s., not significant.
