# Supplementary material for: Two-dimensional NIR-II AIE nanotheranostic probes with ultralarge Stokes shifts for surgical navigation and ablation of glioma
Source: Sci Adv. 2026 Mar 6;12(10):eaeb5389. doi: 10.1126/sciadv.aeb5389 (PMC12965306; doi:10.1126/sciadv.aeb5389)
Supplement: Supplementary file 1 — Supplementary Text Figs. S1 to S35 Tables S1 to S6 Legends for movies S1 and S2 [file sciadv.aeb5389_sm.pdf]

Science Advances
AAAS

Supplementary Materials for

Two-dimensional NIR-II AIE nanotheranostic probes with ultralarge Stokes shifts for surgical navigation and ablation of glioma

Yisheng Liu *et al.*

Corresponding author: Ting Han, hanting@szu.edu.cn; Jiefei Wang, jfwang@henu.edu.cn; Bingyang Shi, Bingyang.Shi@uts.edu.au; Ben Zhong Tang, tangbenz@cuhk.edu.cn

Sci. Adv. **12**, eaeb5389 (2026)
DOI: 10.1126/sciadv.aeb5389

The PDF file includes:

Supplementary Text
Figs. S1 to S35
Tables S1 to S6
Legends for movies S1 and S2

Other Supplementary Material for this manuscript includes the following:

Movies S1 and S2

Supplementary Text

Materials information

All chemicals and solvents were used as received without further purification. Ultrapure water (18.2 MΩ·cm) was used for the biological experiments. The Angiopep-2-SH peptide (TFFYGGSRGKRNNFKTEEYC, purity >98%) was purchased from ChinaPeptides Co., Ltd. (Shanghai, China). 1,2-Distearoyl-sn-glycerol-3-phosphoethanolamine-N-[methoxy (polyethylene glycol)-2000] (DSPE-PEG_{2000}, purity 99%) and DSPE-PEG_{2000}-COOH (purity >95%) were obtained from Xi'an Ruixi Biological Technology Co., Ltd. (Shanxi, China). Poly(ethylene oxide)-poly(propylene oxide)-poly(ethylene oxide) triblock copolymer (P123) and poly(ethylene oxide)-poly(propylene oxide) triblock copolymer (F127) were purchased from Aladdin Reagent Co., Ltd. (Shanghai, China) and Sigma-Aldrich (St. Louis, MO, USA), respectively. 9,10-Anthracenedipropionic acid (ADPA, purity 95%) was supplied by Ye Yuan Biotechnology Co., Ltd. (Shanghai, China), and Methylene Blue (MB, purity 95%) was purchased from Adamas-beta (Shanghai, China). Cell culture reagents, including high-glucose Dulbecco's Modified Eagle's Medium (DMEM), fetal bovine serum (FBS), and Cell Counting Kit-8 (CCK-8), were supplied by Solarbio Science & Technology Co., Ltd. (Beijing, China). Trypsin-ethylenediaminetetraacetic acid (Trypsin-EDTA, 0.25%, Gibco) was obtained from Thermo Fisher Scientific (Waltham, MA, USA). Fluorescent probes and assay kits including mitochondrial superoxide indicator (MitoSOX™ Red), dihydroethidium (DHE), 2',7'-dichlorodihydrofluorescein diacetate (DCFH-DA), and a terminal deoxynucleotidyl transferase dUTP nick end labeling (TUNEL) apoptosis assay kit were all purchased from Beyotime Biotechnology (Shanghai, China). The primary antibody used was a rabbit monoclonal anti-CD31 (Abcam, 1:100), and the second antibody (1:1000) was purchased from United Experimental & Laboratory Biotechnology Co., Ltd. All other chemicals, including 1-bromopyrene, phenylacetylene, $Pd(PPh_3)_2Cl_2$, CuI, PPh_3, 7,7,8,8-tetracyanoquinodimethane (TCNQ), and 2,3,5,6-tetrafluoro-7,7,8,8-tetracyanoquinodimethane (F_4-TCNQ), were sourced from J&K Scientific Ltd. (Beijing, China).

Synthetic procedures and characterization data for TNQ1 and TNQ2

1-(Phenylethynyl)pyrene was synthesized through the Sonogashira coupling between 1-bromopyrene and phenylacetylene as provided in Fig. S1 according to the previously reported method (*45*).

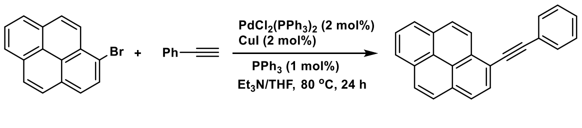

Fig. S1. Synthesis of the starting material. Synthetic route to 1-(phenylethynyl)pyrene.

TNQ1 and TNQ2 were synthesized via the metal-free [2+2] cycloaddition-cycloreversion reaction between alkyne and electron-deficient alkenes F_4-TCNQ and TCNQ as presented in Fig. S2.

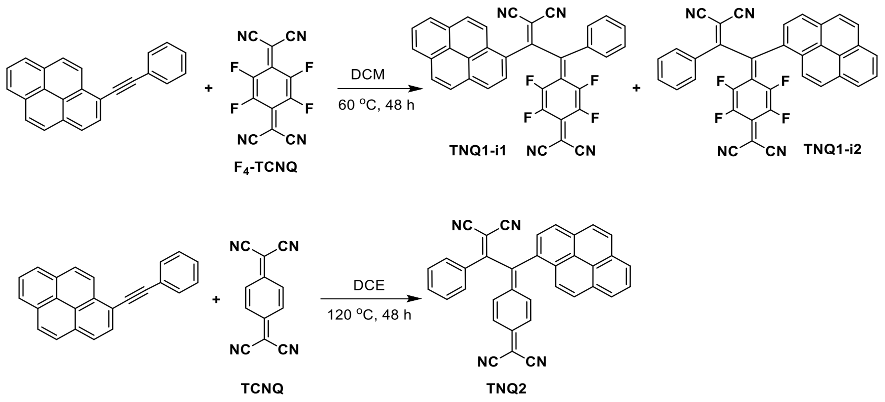

Fig. S2. Synthesis of the target compounds. Synthetic routes to TNQ1 and TNQ2.

Synthesis and characterization of TNQ1

1-(Phenylethynyl)pyrene (0.2 mmol, 60.4 mg) and F_4-TCNQ (60.7 mg, 0.22 mmol) were dissolved in dichloromethane (DCM, 4 mL), and the resulting mixture was stirred at 60 °C for 48 h. Afterward, the solvent was evaporated under reduced pressure, and the crude product was purified by column chromatography using hexane/DCM = 2/1 as the eluent. TNQ1 (black solid, 69 mg, 60%) was obtained as a mixture of two isomers of TNQ1-i1 and TNQ1-i2 that were hard to be separated.

^{1}H NMR (600 MHz, $CDCl_3$) δ 8.35 (d, J = 7.7 Hz, 2H), 8.33–8.30 (m, 4H), 8.29 (d, J = 8.8 Hz, 1H), 8.25–8.21 (m, 4H), 8.16–8.13 (m, 3H), 8.13–8.11 (m, 2H), 8.10–8.05 (m, 4H), 7.85 (d, J = 8.1 Hz, 1H), 7.78 (d, J = 8.2 Hz, 1H), 7.64 (d, J = 7.5 Hz, 2H), 7.57 (d, J = 7.6 Hz, 3H), 7.53 (d, J = 7.5 Hz, 1H), 7.46 (t, J = 7.8 Hz, 2H), 7.36–7.32 (m, 1H), 7.29 (dd, J = 10.4, 4.8 Hz, 3H). **^{13}C NMR** (100 MHz, $CDCl_3$) δ 174.10, 171.63, 135.62, 134.92, 134.79, 133.61, 133.28, 132.90, 132.78, 132.18, 131.81, 131.51, 131.39, 131.27, 131.20, 130.70, 130.34, 130.10, 129.95, 129.52, 129.46, 129.26, 128.55, 128.10, 127.46, 127.36, 127.31, 127.27, 125.50, 125.27, 124.88, 124.57, 124.20, 124.10, 122.50, 122.01, 121.31, 112.90, 112.75, 112.50, 112.31, 112.26, 112.06, 88.79, 86.88. **^{19}F NMR** (376 MHz, $CDCl_3$) δ -127.58, -127.81, -128.25, -128.67, -137.39, -137.49, -137.59, -138.02. **HRMS** calculated for $C_{36}H_{14}F_4N_4$ $[M + H]^+$: 579.1227; found: 579.1228.

Synthesis and characterization of TNQ2

1-(Phenylethynyl)pyrene (0.2 mmol, 60.4 mg) and TCNQ (44.9 mg, 0.22 mmol) were dissolved in DCM (4 mL), and the resulting mixture was stirred at 120 °C for 48 h. Afterward, the solvent was evaporated under reduced pressure, and the crude product was purified by column chromatography using hexane/DCM=2/1 as the eluent. TNQ2 (56 mg, 55%) was eventually obtained as a black solid.

^{1}H NMR (500 MHz, $CDCl_3$) δ 8.28 (dd, J = 14.1, 7.6 Hz, 2H), 8.20 (d, J = 8.9 Hz, 1H), 8.16 (dd, J = 8.7, 1.9 Hz, 2H), 8.10 (d, J = 7.6 Hz, 1H), 8.07 (d, J = 8.9 Hz, 1H), 7.92 (d, J = 9.2 Hz, 1H), 7.80 (d, J = 8.0 Hz, 1H), 7.53 (dd, J = 6.9, 5.5 Hz, 2H), 7.46–7.42 (m, 2H), 7.40–7.37 (m, 1H), 7.36–7.31 (m, 2H), 7.10 (dd, J = 9.8, 1.4 Hz, 1H), 6.86 (dd, J = 9.8, 1.4 Hz, 1H). **^{13}C NMR** (125 MHz, $CDCl_3$) δ 172.39, 153.80, 148.28, 137.40, 135.87, 134.18, 133.54, 133.23, 131.32, 130.83, 130.55, 130.17, 129.58, 129.09, 129.03, 128.22, 127.23, 127.22, 127.14, 127.11, 126.77, 125.16, 124.31, 123.63, 113.26, 113.18, 112.80, 112.75, 88.18, 79.64. **HRMS** calculated for $C_{36}H_{18}N_4$ $[M + H]^+$: 507.1604; found: 507.1597.

Theoretical calculation information

Density functional theory (DFT) calculations of TNQ1 and TNQ2 were carried out by using Gaussian 16 program package. DFT calculations were conducted to optimize the ground-state geometries of TNQ1 and TNQ2 using the PBE1PBE functional and the def2-tzvp basis set. Analytical frequency calculations at the same theoretical levels confirmed that all optimized structures correspond to true energy minima, exhibiting no imaginary frequencies. Subsequently, time-dependent density functional theory (TD-DFT) calculations were performed to investigate the excited-state properties using the same functional and basis set combination (PBE1PBE/def2-tzvp). These calculations provided vertical excitation energy, optimized excited-state geometry, and fluorescence emission energy of TNQ2. This computational framework was further applied to simulate ultraviolet (UV) and photoluminescence (PL) spectra, ensuring the theoretical consistency across ground- and excited-state characterizations.

Molecular dynamics simulations

The molecular geometries of TNQ2 and P123 were optimized using DFT. For molecular dynamics (MD) simulations, two systems were constructed: (1) 10 TNQ2 molecules and (2) 10 TNQ2 + 10 P123 molecules, each solvated in a cubic water box (10 nm edge length). All systems underwent energy minimization via the steepest descent algorithm, followed by a 10 ns NVT equilibration (300 K) to generate amorphous aggregates. These conformations were further energy minimization via the steepest descent method, followed by a 10 ns NPT ensemble simulation (P = 1 atm, T = 300 K) to achieve equilibrium. Production simulations were then conducted under NPT conditions (1 atm, 300 K) for 100 ns to analyze aqueous aggregation behavior. The simulations employed the Parrinello-Rahman barostat and velocity-rescale thermostat for pressure and temperature coupling. Periodic boundary conditions were imposed in all spatial dimensions. The force field parameters for the molecules were derived from the General Amber Force Field (GAFF), and water was modeled using the TIP3P potential. Short-range interactions, including electrostatics and van der Waals forces, were calculated with a cutoff distance of 10 Å. While long-range electrostatics were treated via the Particle Mesh Ewald (PME) method. Hydrogen atoms in all

bonds were constrained using the LINCS algorithm, and a time step of 2 fs was used for all simulations. All simulations were performed using GROMACS 2021.5 (*46*).

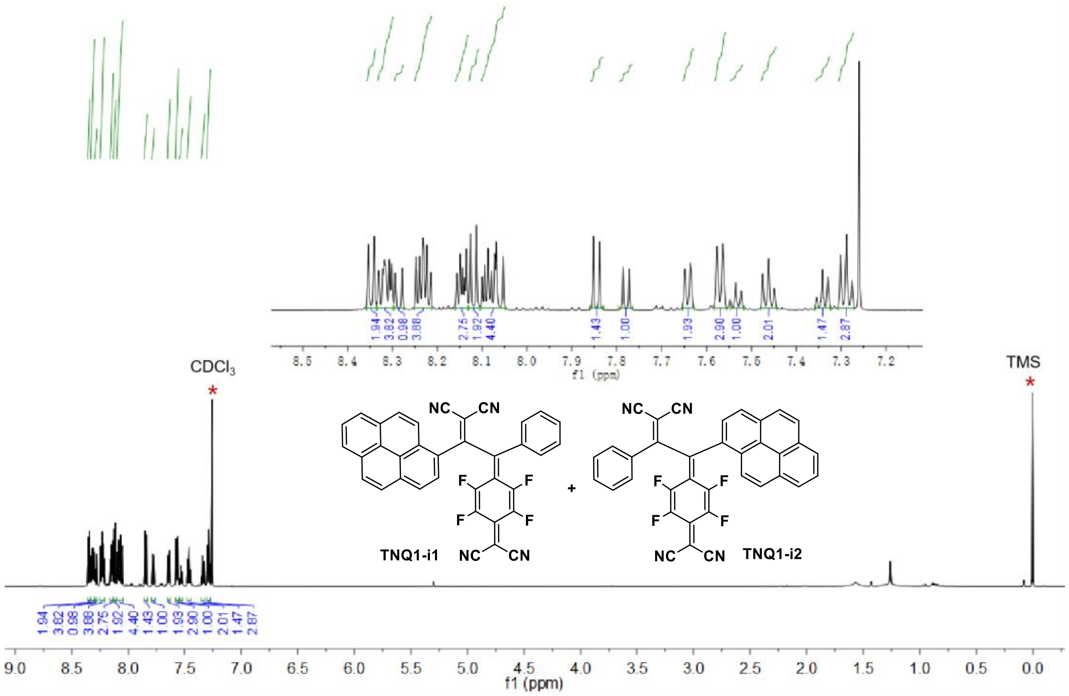

Fig. S3. Characterization of TNQ1 by ^{1}H NMR. The ^{1}H NMR spectrum of TNQ1 in $CDCl_3$. The solvent peaks are marked with asterisks.

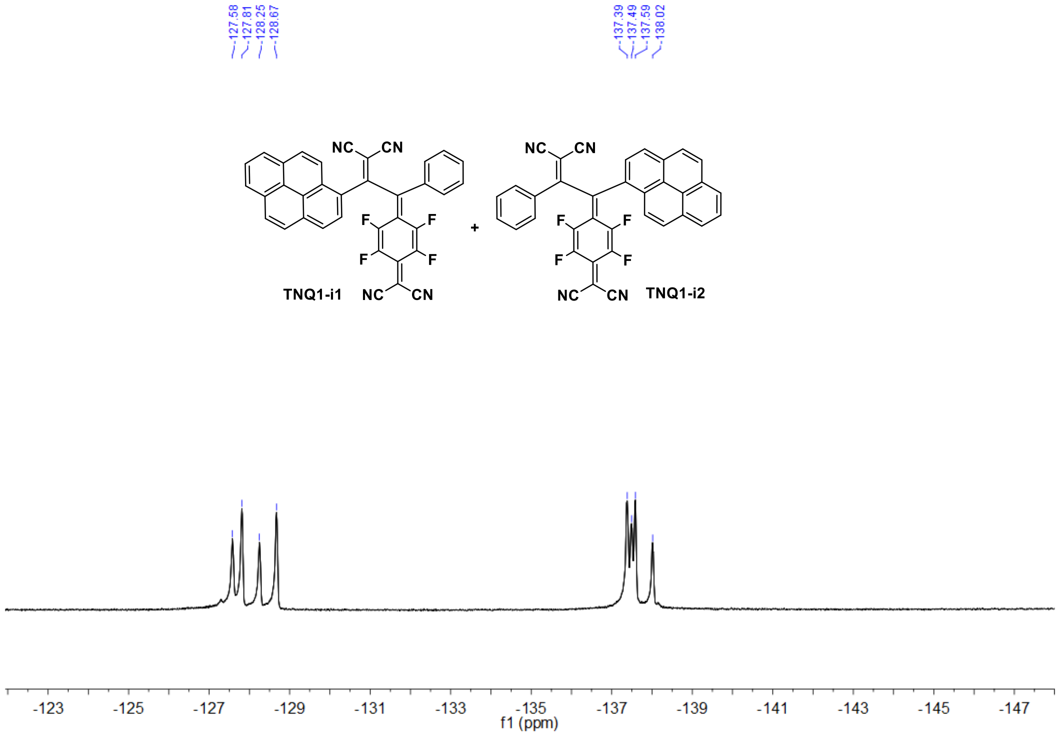

Fig. S4. Characterization of TNQ1 by ^{19}F NMR. The ^{19}F NMR spectrum of TNQ1 in $CDCl_3$.

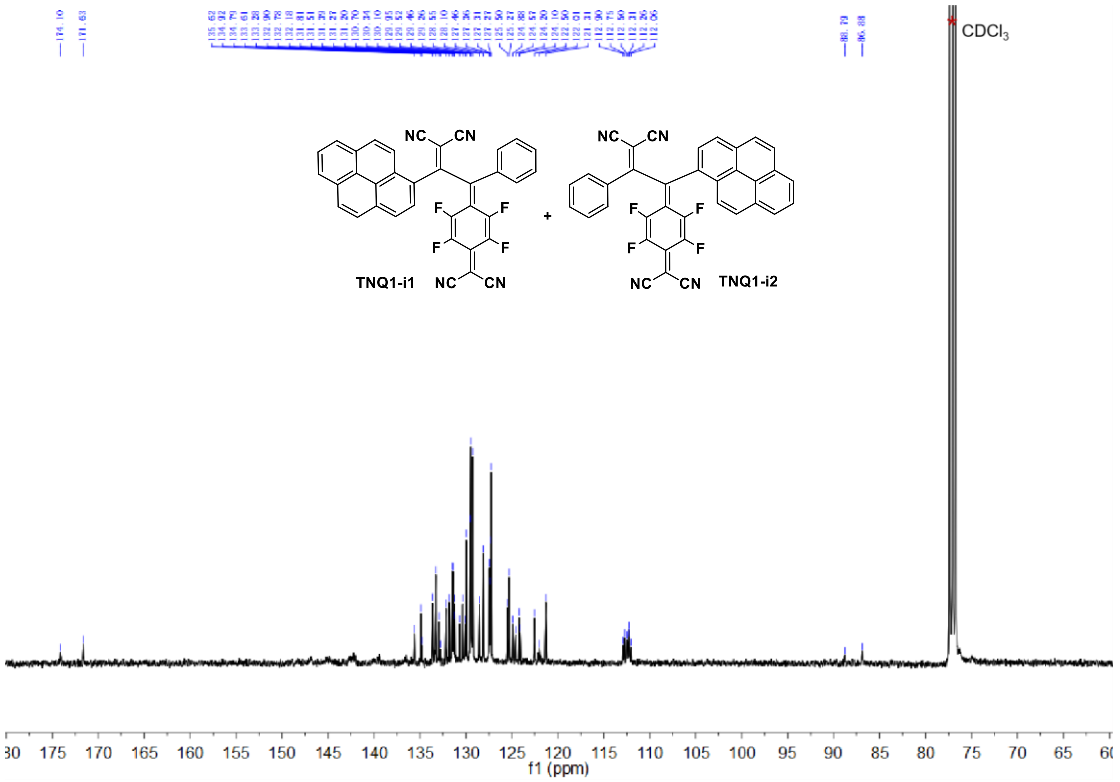

Fig. S5. Characterization of TNQ1 by ^{13}C NMR. The ^{13}C NMR spectrum of TNQ1 in $CDCl_3$. The solvent peaks are marked with asterisks.

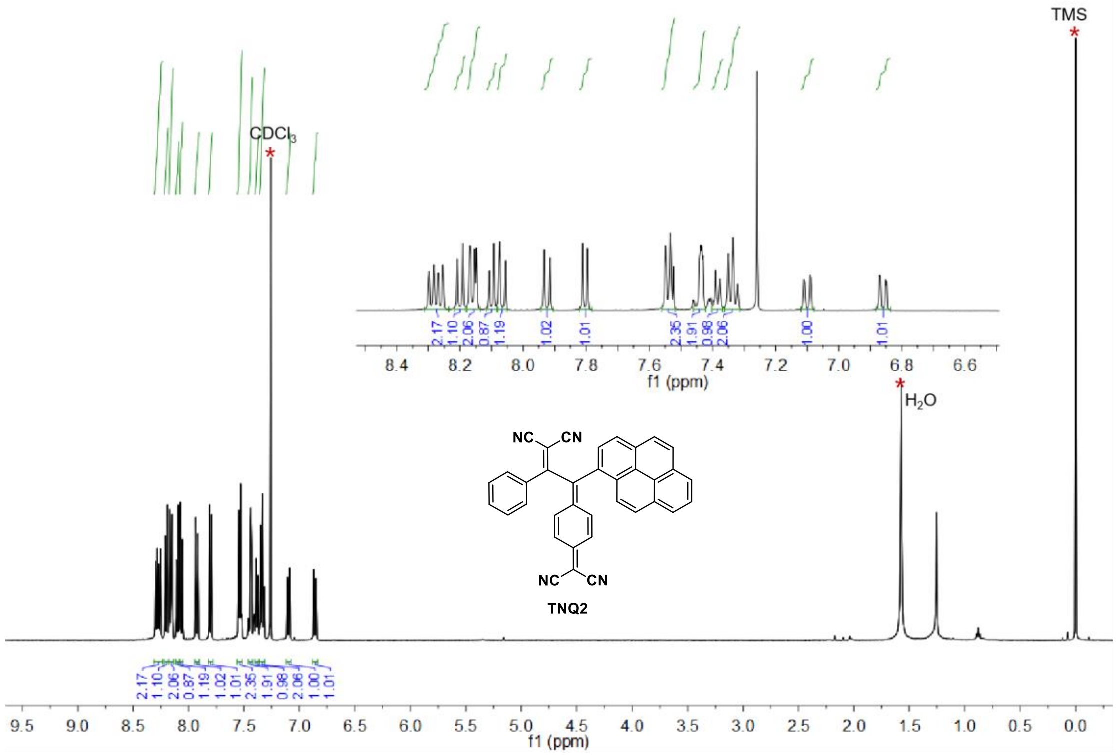

Fig. S6. Characterization of TNQ2 by ^{1}H NMR. The ^{1}H NMR spectrum of TNQ2 in $CDCl_3$. The solvent peaks are marked with asterisks.

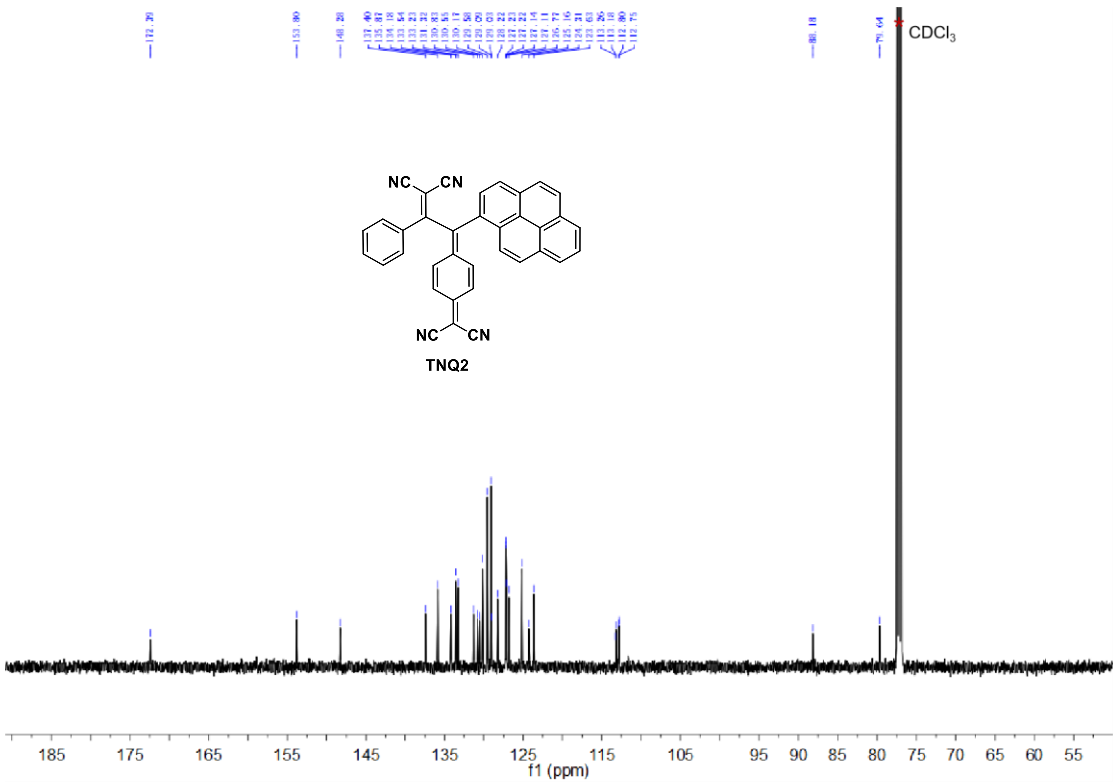

Fig. S7. Characterization of TNQ2 by ^{13}C NMR. The ^{13}C NMR spectrum of TNQ2 in $CDCl_3$. The solvent peaks are marked with asterisks.

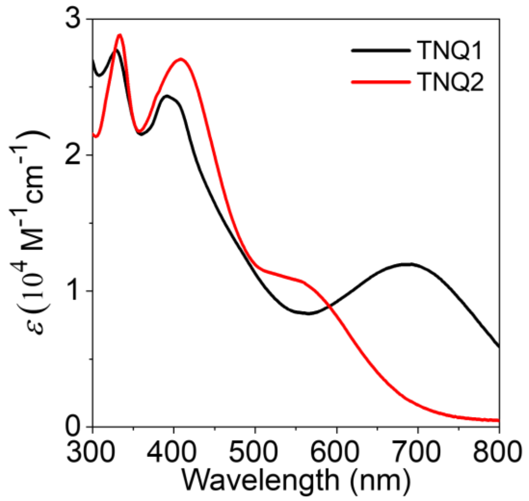

Fig. S8. Comparison of the absorption property. Molar extinction coefficients (ε) from the absorption spectra of TNQ1 and TNQ2 dissolved in tetrahydrofuran solvent.

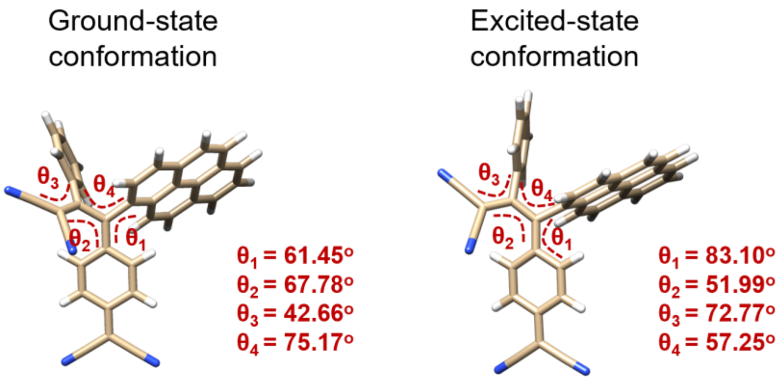

Fig. S9. Optimized ground- and excited-state geometries. The optimized S_0 and S_1 geometries and dihedral angles of TNQ2.

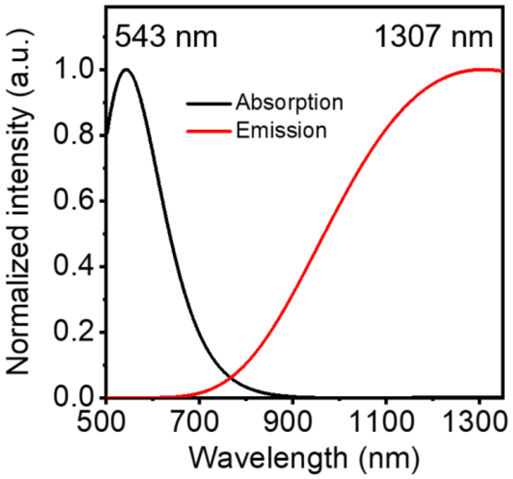

Fig. S10. Simulated absorption and emission spectra. The theoretically simulated absorption and fluorescence spectra of TNQ2.

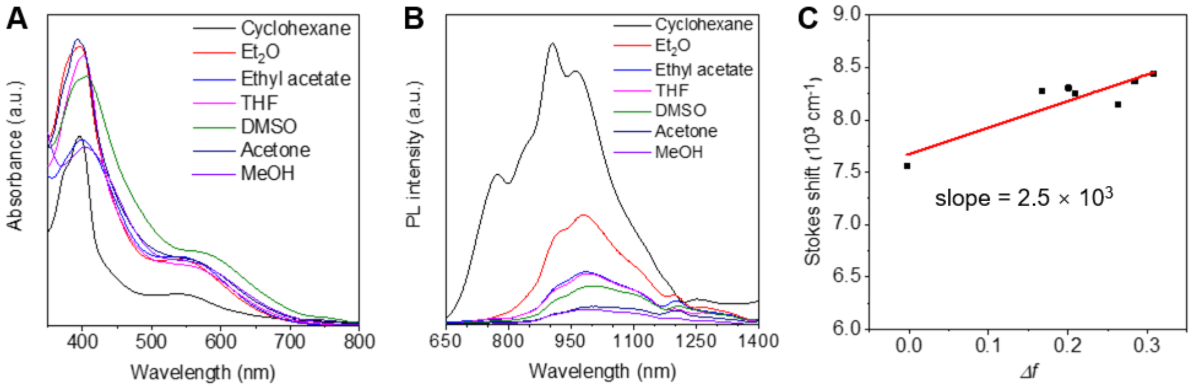

Fig. S11. Study on the solvatochromic effect. (A) Absorption and (B) emission spectra of TNQ2 in various solvents at room temperature. Solution concentration: 100 μM. (C) Lippert-Mataga plot of the relation between Stokes shift ($\Delta\nu$) with solvent polarity Δf for TNQ2.

Lippert-Mataga equation:

$$\Delta\nu = \nu_{\mathrm{abs}} - \nu_{\mathrm{em}} = \frac{2}{hca^3}(\mu_e - \mu_g)^2 \Delta f + const$$

$$\Delta f = \frac{\varepsilon - 1}{2\varepsilon + 1} - \frac{n^2 - 1}{2n^2 + 1}$$

where $\Delta\nu$ is the Stokes shift, h is the Planck constant, c is the speed of light, a is the Onsager cavity radius, μ_e and μ_g refer to the dipolar moments in the excited and ground states, Δf is the solvent polarity function, and ε and n are the dielectric constant and refractive index of the solvent, respectively.

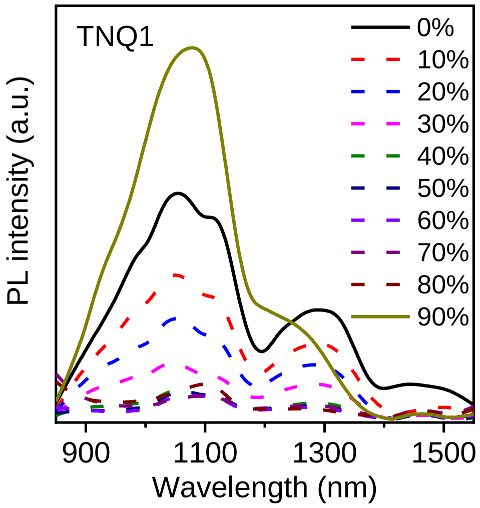

Fig. S12. Fluorescence property of TNQ1 in different states. The PL spectra of TNQ1 in tetrahydrofuran (THF) and THF/water mixtures with different water fractions. Solution concentration: 100 μM; excitation wavelength: 808 nm.

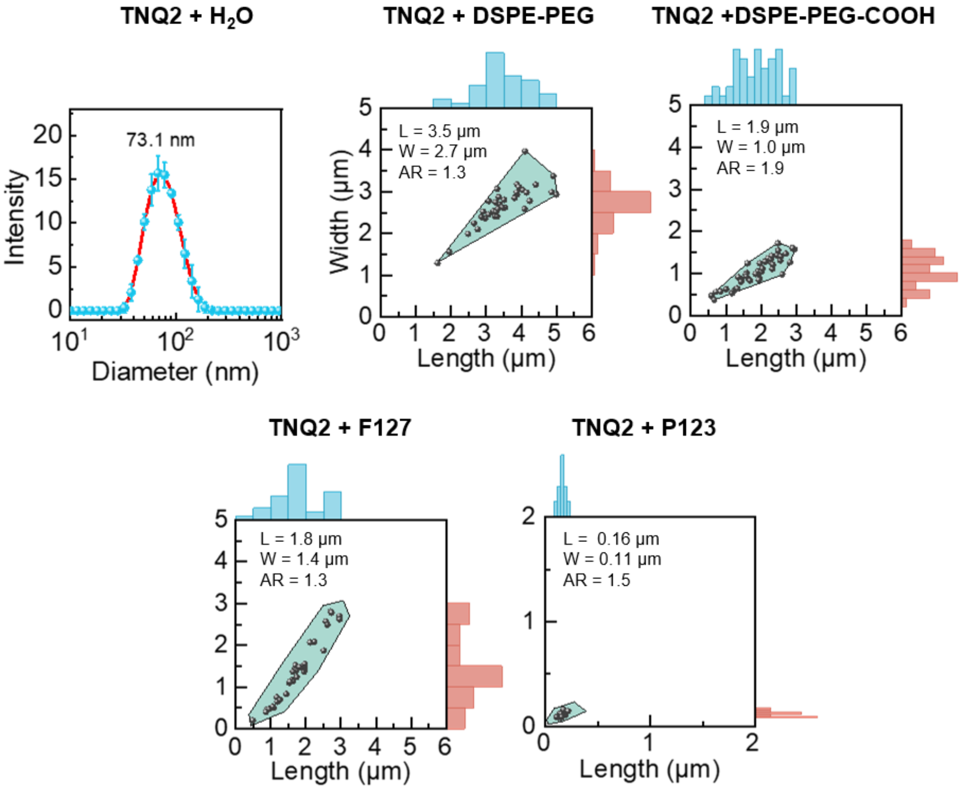

Fig. S13. Surfactant-dependent dimensions of TNQ2 NSs. Statistics of the length and width of nanosheets prepared using different surfactants.

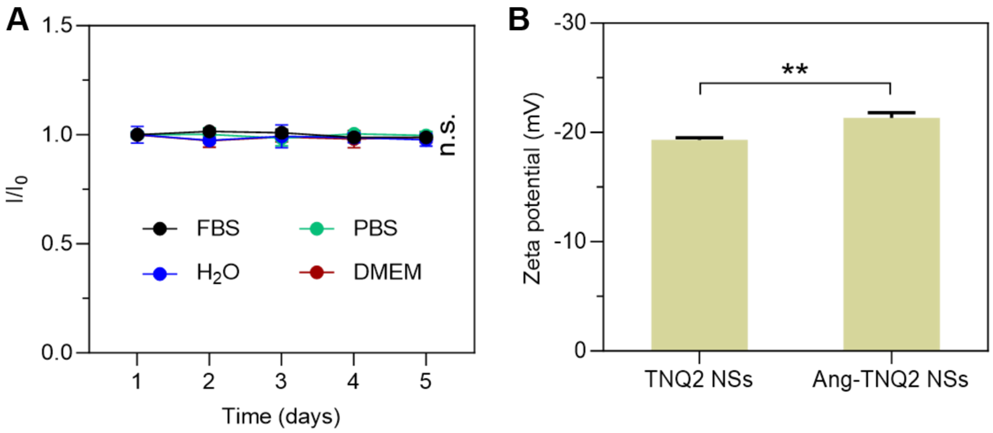

Fig. S14. Structural stability and surface modification of TNQ2 NSs. (A) The particle size changes of TNQ2 NSs in different media over a period of five days (n = 3). (B) The surface potential of TNQ2 NSs before and after Ang-2 modification (n = 3). Data are presented as mean ± SD. Statistical significance was determined by one-way ANOVA for panel (A) and by two-tailed Student's t-test for panel (B). ** $P < 0.01$, n.s., not significant.

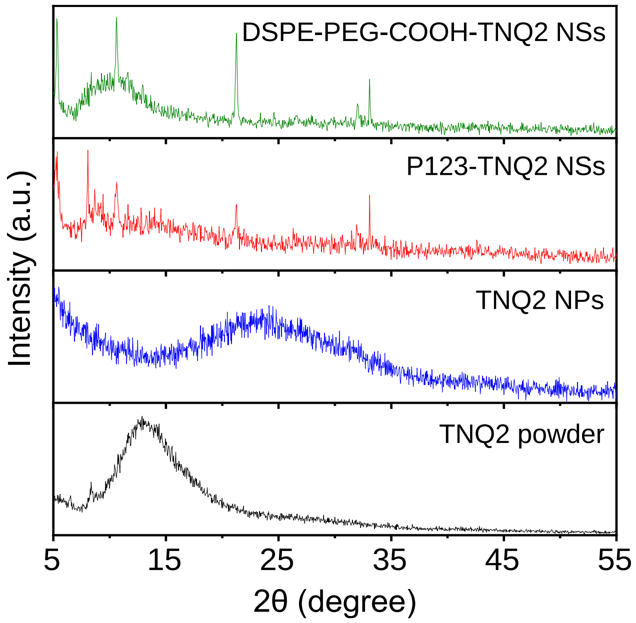

Fig. S15. Molecular packing analysis. X-ray diffraction (XRD) patterns of TNQ2 raw powder, TNQ2 NPs, P123-TNQ2 NSs, and DSPE-PEG-COOH-TNQ2 NSs.

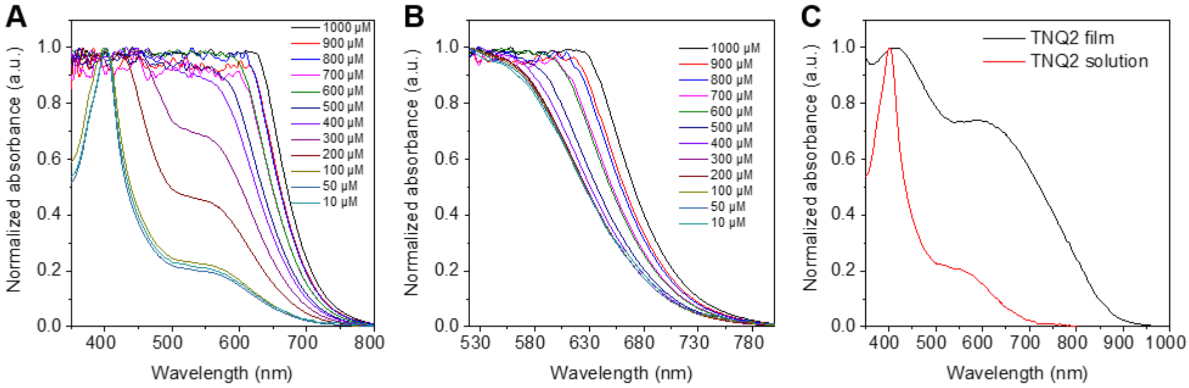

Fig. S16. Concentration-dependent photophysical properties. (A and B) Normalized absorption spectra of TNQ2 in THF solutions with various concentrations. (C) Normalized absorption spectra of TNQ2 in THF solution and in solid thin film state.

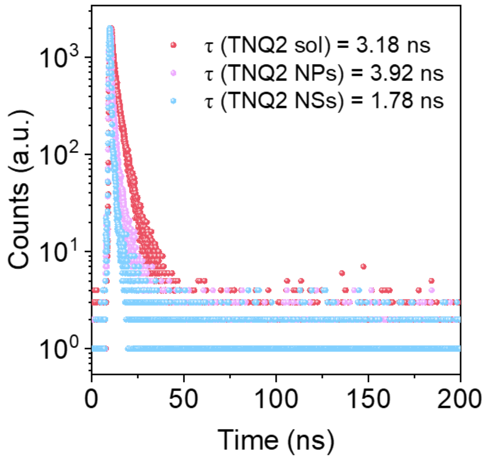

Fig. S17. Fluorescence lifetime in different states. Fluorescence lifetime of TNQ2 in THF solution, TNQ2 NPs and TNQ2 NSs. Molecule concentration: 20 μM, excitation wavelength: 365 nm.

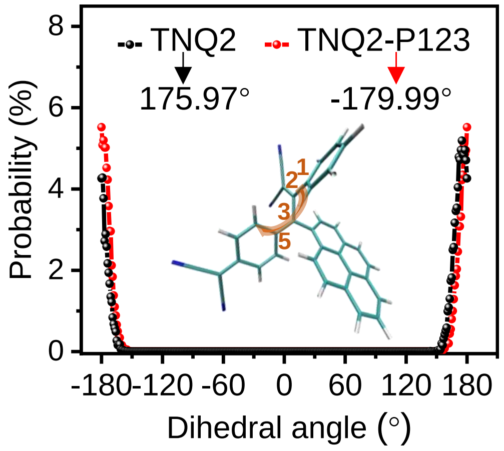

Fig. S18. Theoretically calculated dihedral angle data. Distribution of the dihedral angles between the phenyl and the malononitrile-containing cyclohexadiene plane of the innermost molecule in TNQ2 and TNQ2-P123 aggregates in water.

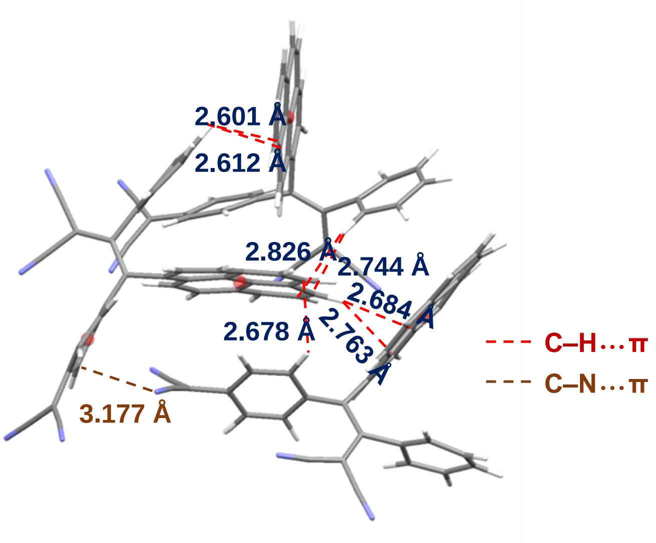

Fig. S19. Packing mode in TNQ2 aggregates. Packing diagram of the intermolecular interactions among TNQ2 molecules in TNQ2 aggregates.

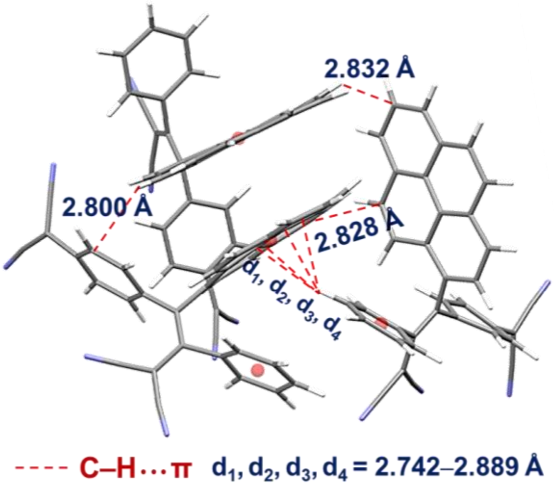

Fig. S20. Packing mode in TNQ2-P123 aggregates. Packing diagram of the intermolecular interactions among TNQ2 molecules in TNQ2-P123 aggregates.

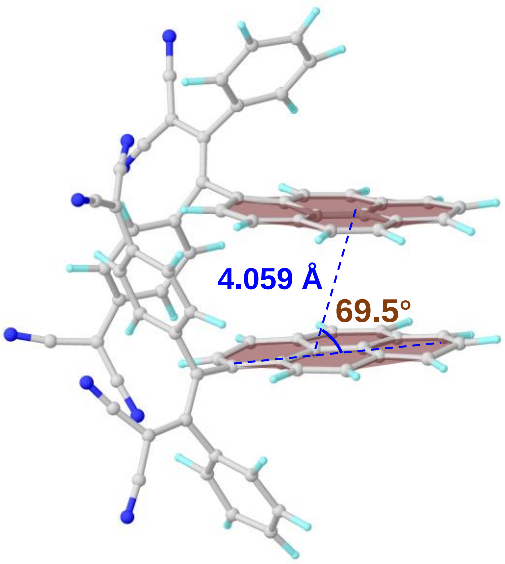

Fig. S21. H-aggregates in TNQ2-P123. Packing diagrams showing the formation of H-aggregates among TNQ2 molecules in TNQ2-P123 aggregates.

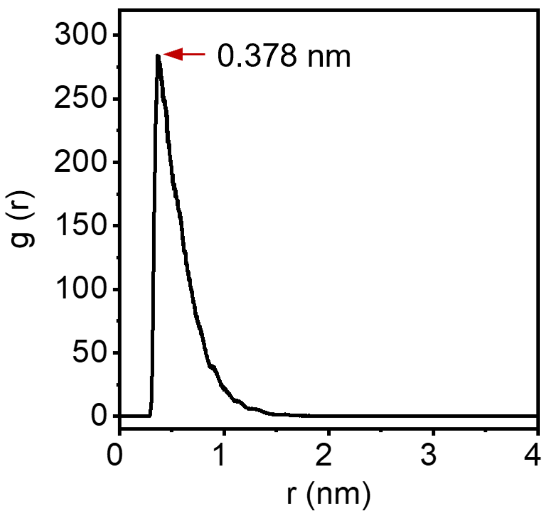

Fig. S22. Calculation on radial distribution function. Radial distribution function of TNQ2 molecules in TNQ2-P123 aggregates.

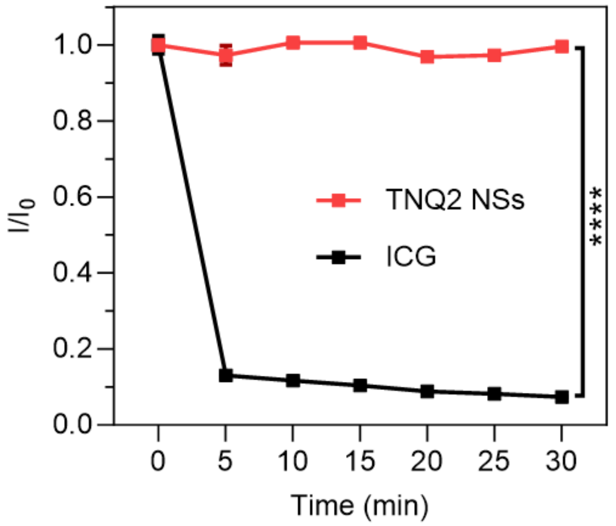

Fig. S23. Evaluation of the anti-photobleaching performance. Absorbance changes of TNQ2 NSs and indocyanine green (ICG) under continuous 808 nm irradiation (1.0 W/cm²) for 30 min (n = 4). Data is presented as mean ± SD. Statistical significance was determined by the two-tailed Student's t-test. **** $P < 0.0001$.

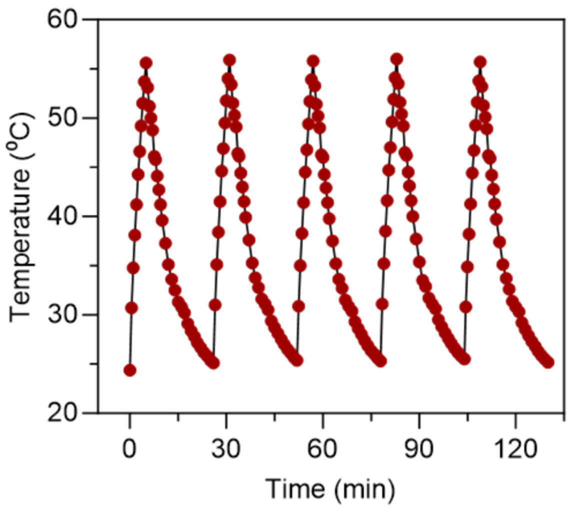

Fig. S24. Evaluation of the photothermal stability. The photothermal stability of TNQ2 NSs (400 μg/mL) over five consecutive laser on/off cycles upon 808 nm irradiation (1.0 W/cm^2).

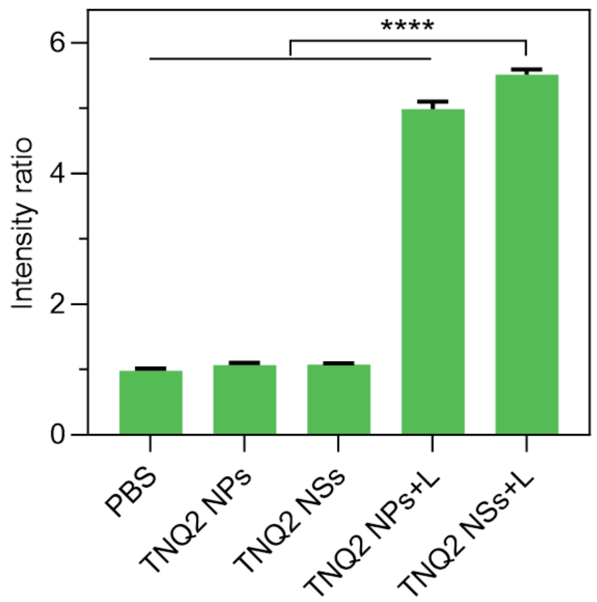

Fig. S25. Assessment of photo-induced reactive oxygen species (ROS). ROS of TNQ2 NSs and TNQ2 NPs with and without 808 nm irradiation (1.0 W/cm^2, termed as L) for 5 min (n = 3). Data are presented as mean ± SD. Statistical significance was determined by one-way ANOVA. **** P < 0.0001.

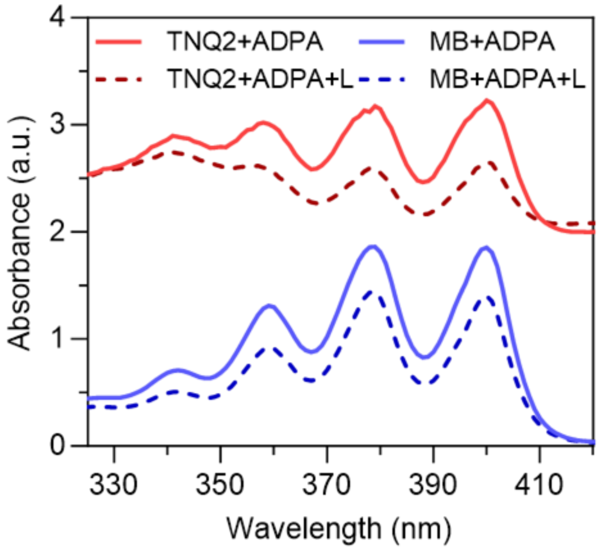

Fig. S26. Comparative evaluation of singlet oxygen generation capability. Absorption spectra of ADPA (0.1 M) in the presence of either TNQ2 NSs (50 µM) or MB (50 µM), before and after 635 nm irradiation (0.5 W/cm² for 5 min).

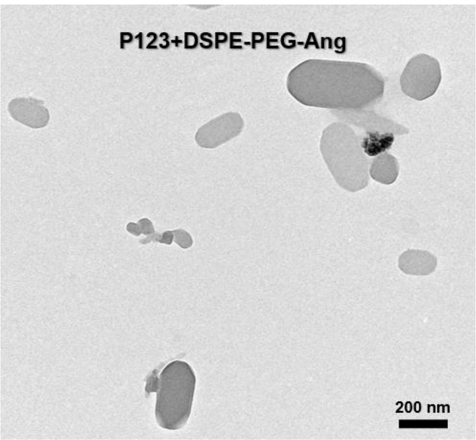

Fig. S27. Morphological characterization of nanosheets. Transmission electron microscopy image of Ang-TNQ2 nanosheets prepared by DSPE-PEG-Ang.

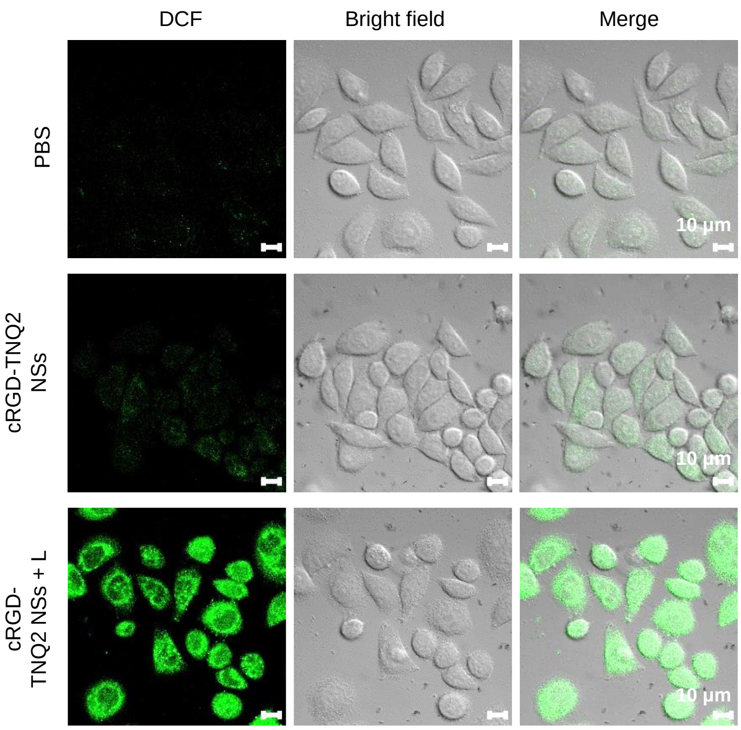

Fig. S28. Intracellular photo-induced ROS generation of NSs. ROS detection in EC109 cells using green DCFH-DA probe.

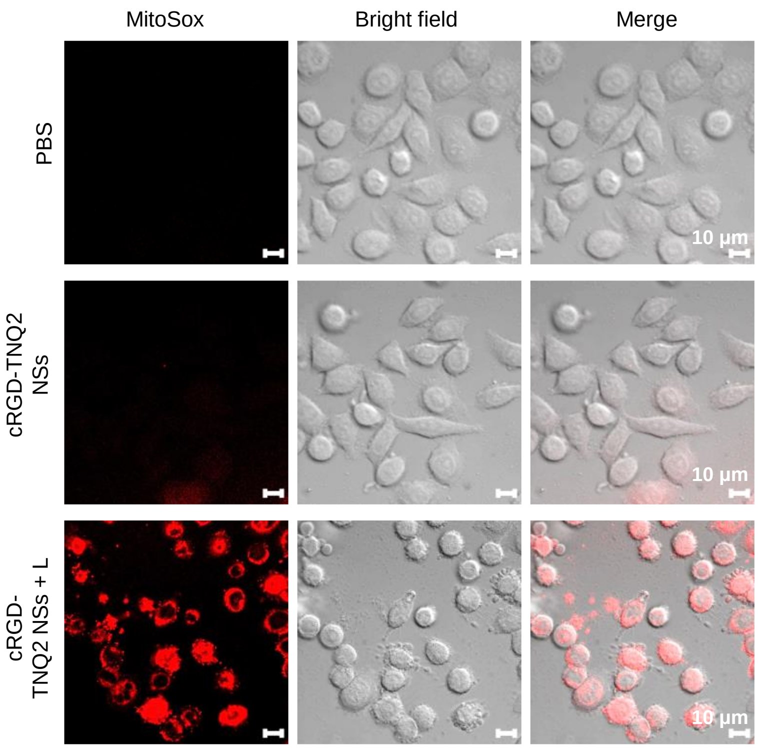

Fig. S29. Detection of photo-induced mitochondrial superoxide generation of NSs. Mitochondrial superoxide detection in EC109 cells detected by MitoSOX Red probe.

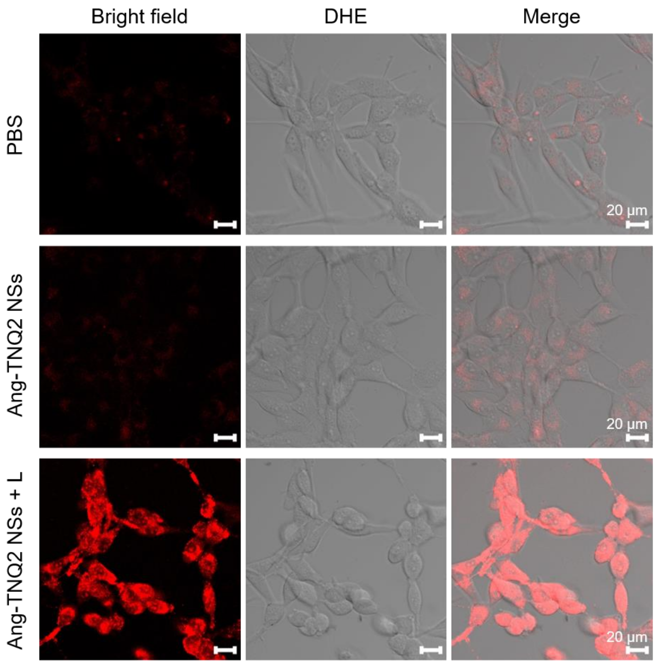

Fig. S30. Detection of photo-induced intracellular superoxide generation of NSs. Confocal images of U87-Luc cells treated with PBS, Ang-TNQ2 NSs (100 μM), and Ang-TNQ2 NSs (100 μM) with 808 nm irradiation (L) at 1.0 W/cm^2 for 5 min. All were stained with DHE before observation.

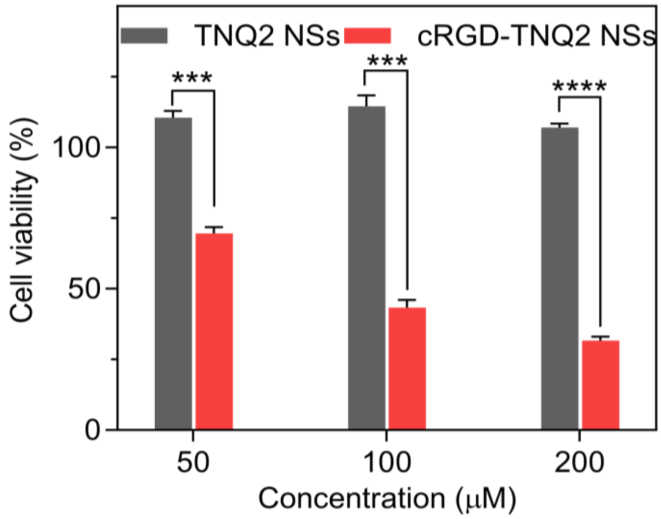

Fig. S31. In vitro phototherapeutic efficacy. Phototherapy efficiency of cyclic Arg-Gly-Asp (cRGD)-modified TNQ2 NSs (cRGD-TNQ2 NSs) and TNQ2 NSs in EC109 cells under 808 nm irradiation (1.0 W/cm², 6 min). Data are presented as mean ± SEM (n = 3). Differences between groups were analyzed by a two-tailed Student's t-test. *** $P < 0.001$, **** $P < 0.0001$.

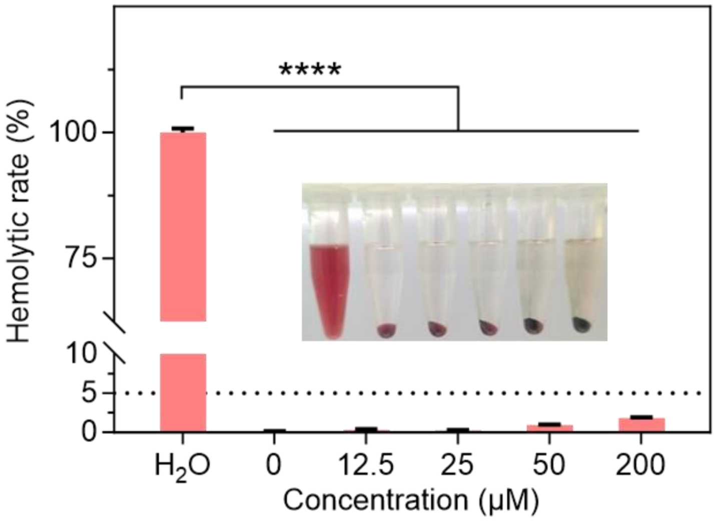

Fig. S32. Biocompatibility assessment of TNQ2 NSs. Hemolysis rate of erythrocytes treated with TNQ2 NSs at varying concentrations (n = 3). Data are presented as mean ± SD. Statistical significance was determined by one-way ANOVA. **** $P < 0.0001$.

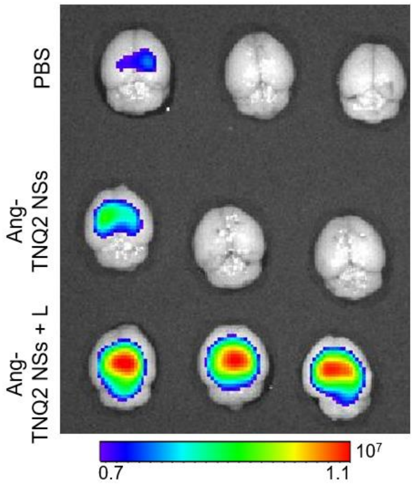

Fig. S33. Visualization of photo-induced ROS in ex vivo brains. Fluorescence imaging of ROS in ex vivo brain (n = 3) from glioma-bearing mice after i.v. injection of PBS, Ang-TNQ2 NSs, or Ang-TNQ2 NSs with 808 nm laser irradiation (0.5 W/cm², 5 min; labeled as “L”).

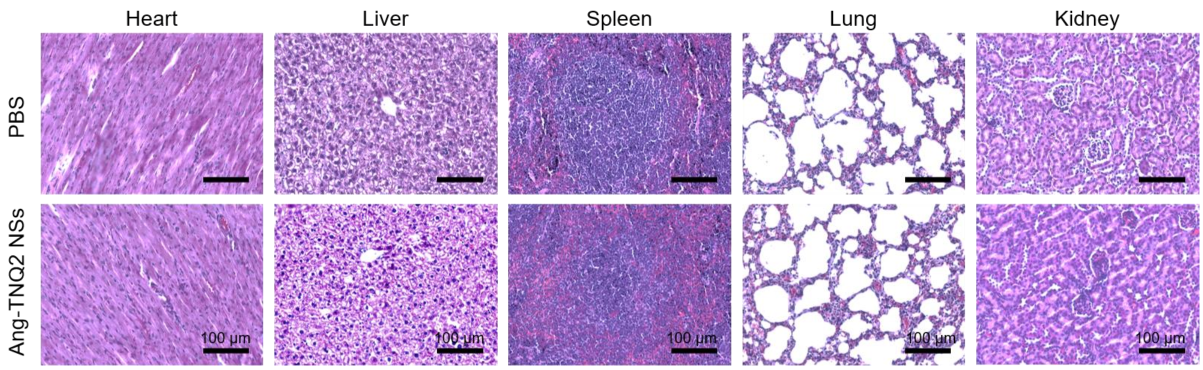

Fig. S34. Histological safety assessment of Ang-TNQ2 NSs. Hematoxylin and Eosin (H&E) staining of major organs (heart, liver, spleen, lung, and kidney) from healthy mice after consecutive intravenous injections of PBS or Ang-TNQ2 NSs (10 mg/kg) 15 times over one month.

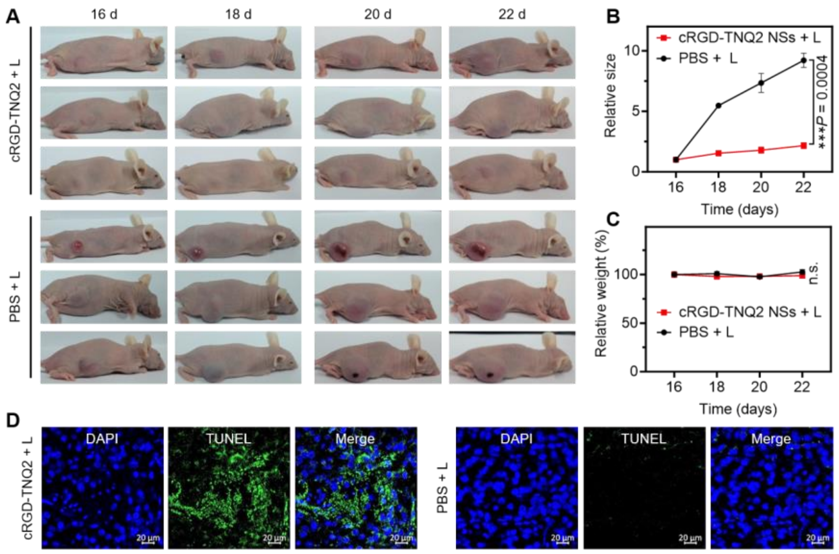

Fig. S35. In vivo phototherapy assessment in subcutaneous EC109 tumor-bearing mice. (**A**) Images monitoring of subcutaneous EC109 tumors in mice (n = 3). (**B** and **C**) Relative tumor size (B) and body weight (C) changes during treatment as a function of time (n = 3). (**D**) Confocal images of TdT-mediated dUTP Nick-End Labeling (TUNEL)-stained tumor sections from the two groups. Data are presented as mean ± SEM. The analytical method employed is the two-tailed Student's t-test. *** $P < 0.001$, n.s., not significant.

Table S1. Ground-state geometry information of TNQ2. Cartesian coordinates for the DFT optimized S_0 geometry (at PBE1PBE/def2-tzvp level) of TNQ2.

Atom	X	Y	Z
C	-0.82081600	3.04070600	3.00743200
C	-1.27181800	4.33351900	2.78191900
C	-0.99019000	4.96471000	1.57871500
C	-0.24750400	4.31595400	0.60838100
C	0.21764100	3.01738200	0.82688400
C	-0.09294600	2.38281200	2.03268400
C	1.00998100	2.29139900	-0.17456400
C	0.70992800	0.85893200	-0.37963400
C	1.96665900	2.89774700	-0.93844300
C	-0.67788200	0.57485600	-0.77916100
C	-1.50144800	-0.37586400	-0.13123300
C	-2.82129600	-0.58820700	-0.61823300
C	-3.30457900	0.14719000	-1.73076700
C	-2.47496500	1.10217100	-2.32100800
C	-1.20224900	1.31722300	-1.84264900
C	-3.67116400	-1.53860500	0.00785200
C	-4.97974400	-1.76470300	-0.49094100
C	-5.42535300	-1.01630500	-1.62155300
C	-4.62473200	-0.09934500	-2.21187500
C	-1.09349300	-1.10539700	1.02906100
C	-1.90898300	-2.00335000	1.62920000
C	-3.22046400	-2.26568200	1.13634200
C	-4.06827500	-3.19850200	1.73531100
C	-5.34139300	-3.41783300	1.23699200
C	-5.79389100	-2.70881300	0.13664200
C	2.38672000	4.24865400	-0.75274600
C	2.62705900	2.22115000	-2.00767700
N	3.16486500	1.71790700	-2.89357000
N	2.77082500	5.32795800	-0.63085200
C	1.68730000	-0.10341900	-0.24597700
C	1.48101700	-1.45078400	-0.71462400
C	2.96138300	0.17286700	0.36946000
C	2.42795200	-2.40062800	-0.58811300
C	3.68233500	-2.11933600	0.05194200
C	3.90106400	-0.78109300	0.52228100
C	4.64174900	-3.10069300	0.20880100
C	5.88331200	-2.82996000	0.83893600
C	4.42457400	-4.42776100	-0.24250900

N	4.23044900	-5.50329400	-0.61168900
N	6.88573100	-2.59509200	1.35919200
H	-1.04068900	2.54167500	3.94370900
H	-1.84954600	4.84708600	3.54163200
H	-1.35308900	5.96843100	1.39220500
H	-0.05055900	4.81289600	-0.33293100
H	0.25678800	1.37263700	2.21062100
H	-2.83886400	1.67164600	-3.16899300
H	-0.57153300	2.05263600	-2.32936200
H	-6.42675700	-1.19609100	-1.99795300
H	-4.97407000	0.46853900	-3.06735500
H	-0.10561900	-0.93502200	1.43575500
H	-1.57030700	-2.54055500	2.50883500
H	-3.71698800	-3.75236800	2.59926300
H	-5.98779300	-4.14717400	1.71111800
H	-6.79247700	-2.88217500	-0.24989800
H	0.55375700	-1.68277100	-1.22197600
H	3.15195500	1.16714800	0.75379700
H	2.26057600	-3.39478700	-0.98611200
H	4.83918900	-0.55005100	1.01330000

Table S2. Excited-state geometry information of TNQ2. Cartesian coordinates for the TD-DFT optimized S_1 geometry (at PBE1PBE/def2-tzvp level) of TNQ2.

Atom	X	Y	Z
C	-2.54985600	3.12793300	1.90132600
C	-3.26072400	3.88854100	0.98526000
C	-2.71481600	4.14800400	-0.26357500
C	-1.46619500	3.64631000	-0.59423400
C	-0.75281200	2.86373300	0.31028600
C	-1.30583600	2.61667000	1.56330000
C	0.60396300	2.34025700	-0.03793900
C	0.73947900	0.97817800	-0.33772000
C	1.62206000	3.30759700	-0.11913500
C	-0.49686300	0.31183000	-0.86229500
C	-1.32489700	-0.51463000	-0.05063600
C	-2.48432200	-1.11276000	-0.59898200
C	-2.82457900	-0.89434600	-1.95271100
C	-1.96927100	-0.09583900	-2.74030600
C	-0.83124500	0.47062300	-2.20608000
C	-3.30104500	-1.94017000	0.19878800
C	-4.46478100	-2.52970800	-0.34738600
C	-4.79220700	-2.28267600	-1.69917300
C	-3.98984800	-1.48961200	-2.48002800
C	-0.98770900	-0.79690500	1.28852100
C	-1.77710600	-1.61152400	2.06612700
C	-2.95067400	-2.19302200	1.54609100
C	-3.77934400	-3.02944300	2.32545000
C	-4.92147600	-3.59787600	1.79192500
C	-5.26326100	-3.35422500	0.47256300
C	1.42068000	4.61225800	0.39194100
C	2.82877800	3.10827500	-0.82531000
N	3.80197300	2.96352300	-1.43185600
N	1.26915600	5.67801700	0.81326000
C	1.91864800	0.17636600	-0.27152000
C	1.98270900	-1.09474100	-0.90306800
C	3.04282700	0.53342000	0.52071500
C	3.06621200	-1.91411200	-0.78737800
C	4.18099500	-1.54842600	0.00946000
C	4.11723700	-0.29208200	0.66547100
C	5.28093500	-2.41445000	0.16697900
C	6.37501100	-2.07280000	0.98794000
C	5.31188200	-3.67118000	-0.47163500
N	5.30246600	-4.69796500	-1.00423000

N	7.25833500	-1.77751200	1.67340700
H	-2.95767400	2.94533300	2.88915100
H	-4.23040400	4.29411800	1.25033200
H	-3.25560600	4.75913100	-0.97711200
H	-1.02604900	3.87610100	-1.55842100
H	-0.73889900	2.04654500	2.29086700
H	-2.22197600	0.07351200	-3.78153000
H	-0.18558800	1.07756000	-2.82823400
H	-5.68481100	-2.73463800	-2.11651900
H	-4.24434600	-1.31478700	-3.51932000
H	-0.08084500	-0.36791200	1.69249400
H	-1.49739600	-1.81555900	3.09339700
H	-3.50428800	-3.22321900	3.35615500
H	-5.54618000	-4.23592100	2.40400300
H	-6.15722800	-3.80102600	0.05166000
H	1.15790800	-1.42918800	-1.52061200
H	3.03203200	1.46911600	1.06293400
H	3.08120200	-2.86719900	-1.30367300
H	4.94133500	0.00877900	1.30218000

Table S3. Photophysical parameters of TNQ2 in different states. Summary table of the photophysical parameters of the THF solution, nanoparticles, and nanosheets of TNQ2.

	Φ^a (%)	τ^a (ns)	k_r (10^7 s^{-1})^b	k_{nr} (10^7 s^{-1})^c
TNQ2 solution	0.02	3.18	0.007	31.440
TNQ2 NPs	0.3	3.92	0.077	25.434
TNQ2 NSs	0.3	1.78	0.169	56.011

^{a} Φ = absolute fluorescence quantum yield; τ = fluorescence lifetime. ^{b} k_r refers to the radiative transition rate; $k_r = \Phi/\tau$. ^{c} k_{nr} refers to the non-radiative transition rate; $k_{nr} = 1/\tau - k_r$.

Table S4. Simulated energy term between TNQ2 and P123 in TNQ2-P123 aggregate. Energy term of the average interaction energy between TNQ2 and P123 under 100 ns in molecular dynamics simulation for TNQ2-P123 aggregate.

Energy term	Interaction energy between TNQ2 and P123 (kJ/mol)	The proportion of interaction energy between TNQ2 and P123
$E_{\text{vdw-SR}}$	-492.20	84.51%
E_{disper}	-3.79	0.65%
$E_{\text{coulomb-SR}}$	-59.53	10.22%
$E_{\text{coul-recip}}$	-26.89	4.62%
E_{total}	-582.41	100%

Table S5. Simulated energy term between TNQ2 molecules in TNQ2 aggregate. Energy term of the average interaction energy between TNQ2 molecules under 100 ns in molecular dynamics simulation for TNQ2 aggregate.

Energy term	Interaction energy between TNQ2 molecules (kJ/mol)	The proportion of interaction energy between TNQ2 molecules
$E_{\text{vdw-SR}}$	-174.74	85.89%
E_{disper}	-0.60	0.30%
$E_{\text{coulomb-SR}}$	-16.81	8.26%
$E_{\text{coul-recip}}$	-11.30	5.55%
E_{total}	-203.45	100%

Table S6. Simulated energy term between TNQ2 molecules in TNQ2-P123 aggregate. Energy term of the average interaction energy between TNQ2 molecules under 100 ns in molecular dynamics simulation for TNQ2-P123 aggregate.

Energy term	Interaction energy between TNQ2 molecules (kJ/mol)	The proportion of interaction energy between TNQ2 molecules
$E_{\text{vdw-SR}}$	-208.09	75.28%
E_{disper}	-0.60	0.22%
$E_{\text{coulomb-SR}}$	-36.95	13.37%
$E_{\text{coul-recip}}$	-30.78	11.13%
E_{total}	-276.42	100%

Other Supplementary Materials

Movie S1. Movie of simulated TNQ2 aggregates in water. Molecular dynamics simulation movie showing the spatial arrangement and optimized structures of TNQ2 aggregates in water in the absence of P123. Water molecules are indicated by red color.

Movie S2. Movie of simulated TNQ2-P123 aggregates in water. Molecular dynamics simulation movie showing the spatial arrangement and optimized structures of TNQ2 aggregates in water in the presence of P123. P123 molecules are indicated by navy blue color and water molecules are indicated by red color.
